# Improving prime editing with an endogenous small RNA-binding protein

**DOI:** 10.1038/s41586-024-07259-6

**Published:** 2024-04-03

**Authors:** Jun Yan, Paul Oyler-Castrillo, Purnima Ravisankar, Carl C. Ward, Sébastien Levesque, Yangwode Jing, Danny Simpson, Anqi Zhao, Hui Li, Weihao Yan, Laine Goudy, Ralf Schmidt, Sabrina C. Solley, Luke A. Gilbert, Michelle M. Chan, Daniel E. Bauer, Alexander Marson, Lance R. Parsons, Britt Adamson

**Affiliations:** 1 Department of Molecular Biology, Princeton University, Princeton, NJ USA; 2 Lewis–Sigler Institute for Integrative Genomics, Princeton University, Princeton, NJ USA; 3 Gladstone–UCSF Institute of Genomic Immunology, San Francisco, CA USA; 4 Division of Hematology/Oncology, Boston Children’s Hospital, Boston, MA USA; 5 Department of Pediatric Oncology, Dana–Farber Cancer Institute, Boston, MA USA; 6 Harvard Stem Cell Institute, Cambridge, MA USA; 7 Broad Institute, Cambridge, MA USA; 8 Department of Pediatrics, Harvard Medical School, Boston, MA USA; 9 Department of Chemistry, Princeton University, Princeton, NJ USA; 10 Biomedical Sciences Graduate Program, University of California, San Francisco, San Francisco, CA USA; 11 Arc Institute, Palo Alto, CA USA; 12 Department of Laboratory Medicine, Medical University of Vienna, Vienna, Austria; 13 Department of Urology, University of California, San Francisco, San Francisco, CA USA; 14 Helen Diller Family Comprehensive Cancer Center, University of California, San Francisco, San Francisco, CA USA; 15 Innovative Genomics Institute, University of California, Berkeley, Berkeley, CA USA; 16 Department of Medicine, University of California, San Francisco, San Francisco, CA USA; 17 Parker Institute for Cancer Immunotherapy, San Francisco, CA USA; 18 Present Address: Immunology and Microbial Pathogenesis Program, Weill Cornell Graduate School of Medical Sciences, New York, NY USA

**Keywords:** Functional genomics, Functional genomics, Gene therapy

## Abstract

Prime editing enables the precise modification of genomes through reverse transcription of template sequences appended to the 3′ ends of CRISPR–Cas guide RNAs^1^. To identify cellular determinants of prime editing, we developed scalable prime editing reporters and performed genome-scale CRISPR-interference screens. From these screens, a single factor emerged as the strongest mediator of prime editing: the small RNA-binding exonuclease protection factor La. Further investigation revealed that La promotes prime editing across approaches (PE2, PE3, PE4 and PE5), edit types (substitutions, insertions and deletions), endogenous loci and cell types but has no consistent effect on genome-editing approaches that rely on standard, unextended guide RNAs. Previous work has shown that La binds polyuridine tracts at the 3′ ends of RNA polymerase III transcripts^2^. We found that La functionally interacts with the 3′ ends of polyuridylated prime editing guide RNAs (pegRNAs). Guided by these results, we developed a prime editor protein (PE7) fused to the RNA-binding, N-terminal domain of La. This editor improved prime editing with expressed pegRNAs and engineered pegRNAs (epegRNAs), as well as with synthetic pegRNAs optimized for La binding. Together, our results provide key insights into how prime editing components interact with the cellular environment and suggest general strategies for stabilizing exogenous small RNAs therein.

## Abstract (web summary)

Genome-scale genetic screens identify the small RNA-binding protein La as a strong mediator of prime editing.

## Main

Efforts to repurpose CRISPR–Cas systems have produced a suite of genome-editing tools, including programmable nucleases, base editors and prime editors^3^. Prime editors use reverse transcription to install different types of edits into genomes with minimal unwanted mutational by-products^4^. Compared with other approaches, prime editing is precise and highly versatile. The approach has therefore been adopted for diverse applications (for example, genetic modelling, functional genomics and development of genetic medicines)^1^. Numerous studies have also sought to build enhanced prime editing systems, with a major focus on improving editing efficiency, which is typically low and highly variable^1,4^. However, much remains unknown about how prime editing works and how interactions with the cellular environment affect editing outcomes.

**Fig. 1:**
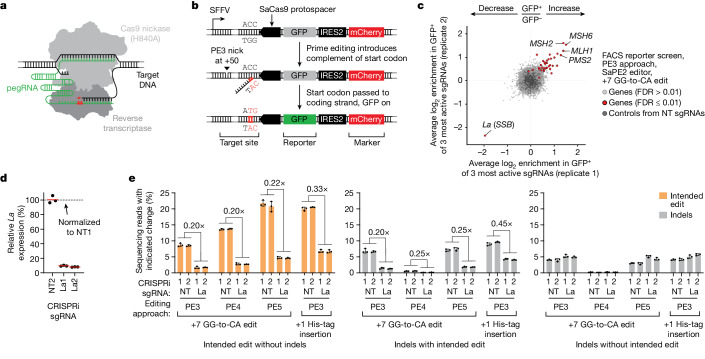
Genome-scale CRISPRi screens identify La as a key determinant of prime editing. **a**, Schematic of prime editing. **b**, Schematic of the FACS reporter of prime editing. **c**, Gene-level phenotypes from genome-scale CRISPRi screen performed in FACS reporter cells with the SaPE2 editor, +7 GG-to-CA edit and the PE3 approach. Phenotypes represent enrichment of normalized sgRNA counts in GFP^+^ over GFP^–^ populations after prime editing (average for the top three sgRNAs per gene). Hit genes (FDR ≤ 0.01) were identified using CRISPhieRmix^16^. Pseudogene controls generated from randomly selected non-targeting (NT) sgRNAs. **d**, Quantification of CRISPRi-mediated *La* depletion. Reverse transcription followed by quantitative PCR (RT–qPCR) of RNA from K562 CRISPRi cells with integrated MCS reporter. Data are normalized to *ACTB* and are presented relative to a non-targeting sgRNA (NT1). La1 and La2, *La*-targeting sgRNAs. **e**, Percentages of prime editing outcomes produced at the integrated MCS reporter using the SaPE2 editor with or without depletion of *La* in K562 CRISPRi cells. Percentages of intended prime editing without indels (left), indels with the intended prime edit (middle) and indels without the intended edit (right) plotted separately. Editing components delivered by plasmid transfection in **c** and **e**. Horizontal bars in **d** indicate geometric means (*n* = 3 independent biological replicates). Data and error bars in **e** indicate mean ± s.d. (*n* = 3 independent biological replicates). Image of the prime editor protein in **a** adapted from ref. ^5^, Elsevier, under a Creative Commons licence CC BY 4.0. Images of DNA and pegRNA in **a** adapted from ref. ^40^, Elsevier.

Prime editors minimally consist of an engineered Cas9 protein (Cas9 H840A nickase fused to a reverse transcriptase) and a pegRNA that specifies both the DNA target and the intended edit^4^ (Fig. 1a). To install the edit, the prime editor protein binds the pegRNA and, directed by the spacer sequence of that pegRNA, finds a complementary DNA target. Once bound to the target, the editing complex nicks a displaced DNA strand and releases a 3′ DNA end. This end can then hybridize to the 3′ extension of the pegRNA and prime reverse transcription of the pegRNA-encoded edit, which is ultimately incorporated into the genome or removed by DNA mismatch repair (MMR)^5,6^.

Several features that affect prime editing efficiency have already been reported, including the expression, stability, localization and activity of editing components, and the chromatin context of targeted loci^1,7^. We have also previously shown that small prime edits can be installed with increased efficiency when MMR is suppressed or evaded^5^. That study provided a clear example of how mechanistic understanding can contribute to technological improvement. To identify additional cellular determinants of prime editing, here we performed genome-scale, CRISPR-interference (CRISPRi) screens, from which we identified a key mediator: the small RNA-binding protein La. Subsequent characterization of La then revealed a functional interaction with pegRNAs, which we exploited to substantially enhance prime editing efficiency.

## CRISPRi screens identify prime editing determinants

Genetic screens have been used to study prime editing^5–7^, but such efforts have interrogated only genes associated with DNA repair processes. Given this limitation, we sought to perform genome-scale screens—which have yet to be realized for this or any other CRISPR-based genome-editing technology^5–10^. To enable screening, we developed a reporter system in which installation of an intended prime edit switches on a reporter gene (Fig. 1b). By design, this system transcribes a single bicistronic mRNA but, owing to lack of a properly positioned start codon (ATG), produces only a constitutive marker protein driven by an internal ribosome entry site (IRES)^11^, until an in-frame ATG is installed at a defined target site by prime editing. Once installed, this ATG induces translation of a second upstream gene, thus producing an easily measurable readout of intended prime edit installation. To enable this reporter system to be paired with CRISPRi, which relies on *Streptococcus pyogenes* Cas9 (SpCas9)^12–14^, we included two protospacers in the target site for use with an orthogonal *Staphylococcus aureus* Cas9 (SaCas9)-based prime editor (SaPE2)^5^: one for ATG installation and another at which a +50 complementary strand nick can be introduced. Such nicks can enhance prime editing efficiency, and their inclusion, through the use of additional single guide RNAs (sgRNAs), constitutes the PE3 approach^4^. Editing without such nicks is called the PE2 approach.

**Extended Data Fig. 1:**
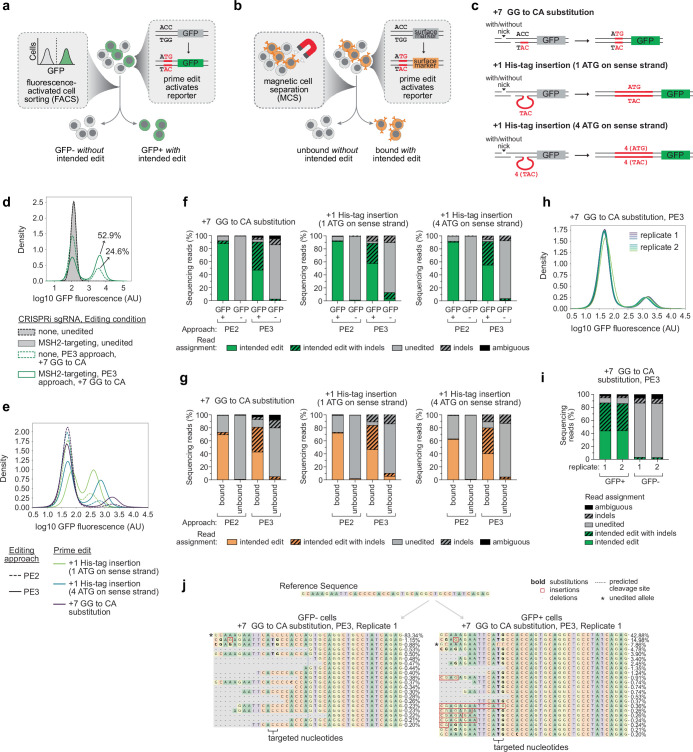
Characterization of prime editing reporters before and during genome-scale CRISPRi screens. **a**, Schematic of isolating prime edited cells with intended edit using our FACS reporter. This reporter expresses GFP upon installation of select prime edits, thus enabling separation of cells into mostly edited or mostly unedited populations using flow cytometry. The complete FACS reporter is depicted in Fig. 1b. **b**, Schematic of isolating prime edited cells with intended edit using our MCS reporter. This reporter expresses a synthetic cell surface marker (Igκ-hIgG1-Fc-PDGFRβ^15^) upon installation of select prime edits, thus enabling separation of cells into mostly edited or mostly unedited populations using magnetic Protein G beads. The complete MCS reporter is depicted in Fig. 2f. **c**, Three prime edits capable of ‘switching on’ our FACS and MCS reporters (depicted with the former). **d**, Flow cytometry analysis of GFP expression in our FACS reporter cells (K562 CRISPRi cells with stably integrated FACS reporter) with and without prime editing (SaPE2, +7 GG to CA, PE3 with a + 50 complementary strand nick), and with and without transduction of an MSH2-targeting sgRNA. **e**, Flow cytometry analysis of GFP expression in our FACS reporter cells after prime editing with each of the edits depicted in c. **f**, Percentages of prime editing outcomes in GFP+ or GFP- cells isolated by FACS after prime editing with each of the edits depicted in c. Outcomes quantified by sequencing the FACS reporter target site. Flow cytometry analysis of edited cell populations prior to sorting presented in e. **g**, Percentages of prime editing outcomes in MCS reporter cells (K562 CRISPRi cells with stably integrated MCS reporter) bound or unbound to Protein G beads after editing with each of the edits depicted in c. Outcomes quantified by sequencing the MCS reporter target site. **h**, Flow cytometry analysis of GFP expression in our FACS reporter cells after transduction with genome-scale CRISPRi library (hCRISPRi-v2) and prime editing with the +7 GG to CA substitution edit. **i**, Percentages of prime editing outcomes observed in GFP+ or GFP- cell population for each replicate of the genome-scale FACS screen. Outcomes quantified by sequencing the FACS reporter target site. **j**, Sequences and frequencies of alleles observed at the FACS reporter target site in cell populations sorted for replicate 1 of the genome-scale FACS screen. Analysis performed with CRISPResso2^47^. Editing components (SaPE2, indicated pegRNAs, nicking sgRNA for PE3) delivered by plasmid transfection in d-j. Data in d-f represent measurements from n = 1 cell populations. Data in g indicate means (n = 3 independent biological replicates). Data in h from n = 4 repeat measurements of each replicate of the genome-scale FACS screen. Data in i represent individual values from each replicate of the genome-scale FACS screen. Data in j depict representative results of n = 2 screen replicates.

We built two versions of our reporter system: one that uses the fluorescent protein eGFP to report on editing and another that uses a synthetic cell surface protein (Igκ-hIgG1-Fc-PDGFRβ)^15^ (Extended Data Fig. 1a,b). These reporter proteins were chosen to facilitate the isolation of edited, reporter-positive cells: GFP through fluorescence-activated cell sorting (FACS) and the surface protein through magnetic cell separation (MCS) with protein G beads. We transduced each reporter construct into a well-established K562 CRISPRi cell line^13,14^ and edited the resulting cells to install one or more start codons (Extended Data Fig. 1c). After editing, our FACS reporter produced distinct populations of GFP^+^ cells (Extended Data Fig. 1d,e). Confirming that the percentages of those GFP^+^ cells reflected intended prime editing efficiencies, depletion of an MMR gene known to suppress small substitution edits (*MSH2*)^5,6^ increased the percentage of GFP^+^ cells (Extended Data Fig. 1d), and PE3-based editing, which is typically more efficient than PE2, produced higher percentages of GFP^+^ cells than PE2-based editing did (Extended Data Fig. 1e). Sequencing target sites from reporter-positive and reporter-negative cells then also confirmed that GFP^+^ FACS reporter cells and protein-G-bead-bound MCS reporter cells were enriched for intended edits (Extended Data Fig. 1f,g).

**Extended Data Fig. 2:**
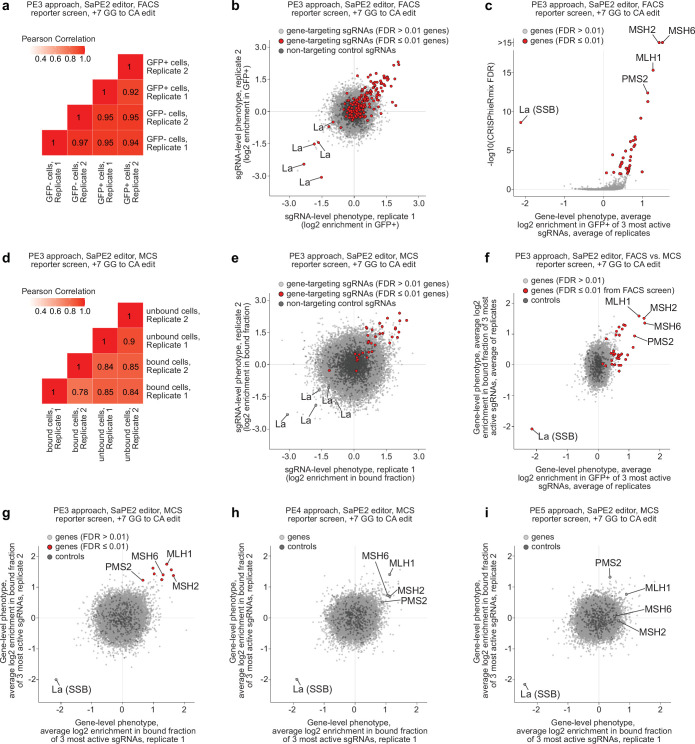
Results of genome-scale CRISPRi screens performed with FACS and MCS reporters. **a**, Pearson correlations of read counts per sgRNA between each pair of samples isolated from the genome-scale FACS screen performed with the PE3 approach. **b**, sgRNA-level phenotypes from each replicate of the genome-scale FACS screen. Phenotypes represent enrichment of normalized sgRNA counts in GFP+ over GFP- populations after prime editing. **c**, Gene-level phenotypes (average of replicates) and per gene FDRs from the genome-scale FACS screen. FDRs determined by CRISPhieRmix^16^. For MSH2 and MSH6, CRISPhieRmix reports an FDR of 0, which we adjusted for plotting. **d**, Pearson correlations of read counts per sgRNA between each pair of samples isolated from the genome-scale MCS screen performed with the PE3 approach. **e**, sgRNA-level phenotypes from each replicate of the genome-scale MCS screen performed with the PE3 approach. Compare to b for screen-to-screen differences in technical variability. **f**, Gene-level phenotypes (average of replicates) from genome-scale FACS and MCS screens performed with the PE3 approach. **g-i**, Gene-level phenotypes from each replicate of MCS reporter screens performed with the PE3 (g), PE4 (h) and PE5 (i) approaches. sgRNAs targeting genes identified as hits (FDR ≤ 0.01, CRISPhieRmix) from the associated screen are indicated in red in b and e. Genes identified as hits (FDR ≤ 0.01, CRISPhieRmix) from the associated screen in c and g and from the FACS screen in f are indicated in red.

Given these results, we proceeded to genome-scale screening. In brief, we transduced our FACS reporter cells with the hCRISPRi-v2 library (18,905 targeted genes, 5 sgRNAs per gene)^14^, introduced prime editing components (SaPE2, +7 GG-to-CA pegRNA, +50 nicking sgRNA) through plasmid transfection and separated the resulting GFP^+^ and GFP^–^ populations. Flow cytometry analyses before sorting confirmed successful editing (Extended Data Fig. 1h), and sequencing of the target site showed expected enrichment of editing outcomes in sorted populations (Extended Data Fig. 1i,j). We then determined the relative enrichment or depletion of each sgRNA across GFP^+^ and GFP^–^ populations by amplicon sequencing (Extended Data Fig. 2a,b and Supplementary Table 1) and calculated gene-level phenotypes (Supplementary Table 2). From this analysis, we identified 36 regulators of prime editing (false discovery rate (FDR) from CRISPhieRmix pipeline^16^ ≤ 0.01) (Fig. 1c and Extended Data Fig. 2c), including only a single positive regulator: the small RNA-binding exonuclease protection factor La (encoded by *SSB*; the alias ‘*La*’ is used here).

Owing to the relative ease of cell separation with our MCS reporter, we also performed several MCS-based, genome-scale screens, specifically using the PE3 approach and two enhanced systems of prime editing called PE4 and PE5, which are PE2 and PE3, respectively, but with the inclusion of a dominant-negative MMR protein (MLH1dn)^5^. Results from these screens were noisier, with higher technical variability (Methods), but reaffirmed several regulators from the FACS screen, including MMR genes (*MSH2*, *MSH6*, *MLH1* and *PMS2*)^5,6^ and ones with unknown roles (*CASP8AP2* and *POLR1D*) (Extended Data Fig. 2d–i and Supplementary Tables 1 and 3). Across all screens, *La* showed the strongest negative phenotype (Fig. 1c and Extended Data Fig. 2c, g–i).

## Loss of La impairs prime editing

**Extended Data Fig. 3:**
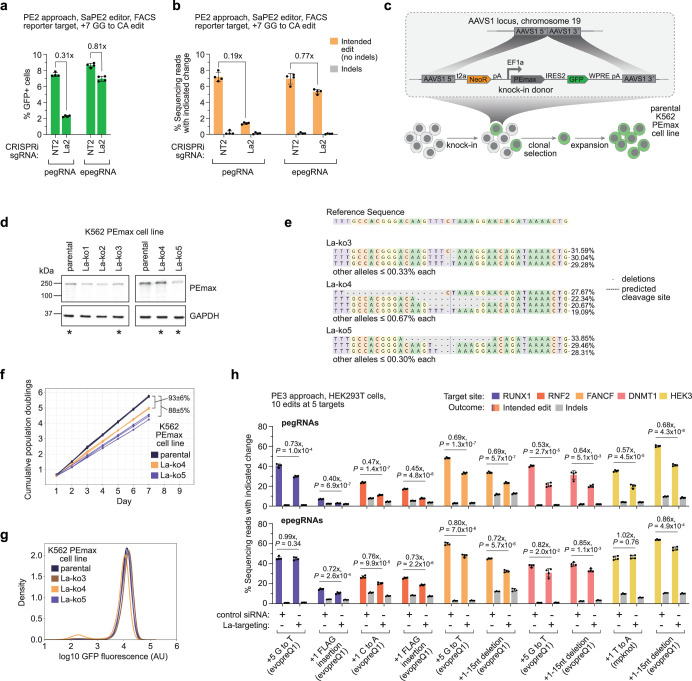
Validating La phenotypes with various genetic perturbation modalities. **a**, **b**, Percentages of prime editing outcomes produced at integrated FACS reporter with pegRNA (left) or epegRNA (right, tevopreQ_1_) in K562 CRISPRi cells after transduction of the indicated sgRNA. Intended editing quantified by flow cytometry (a) or sequencing (b). **c**, Schematic of workflow used to engineer K562 clonal cell lines with PEmax expressed constitutively from the AAVS1 safe-harbor locus (parental K562 PEmax cells). **d**, Western blot analysis of K562 cells constitutively expressing PEmax (K562 PEmax parental) and clones with genetic disruption of La (La-ko1-La-ko5). Asterisks, cell lines used in this study. Images are from the same blot as presented in Fig. 2a. For additional details on imaging, see Methods and Supplementary Fig. 8. **e**, Sequences and frequencies of alleles observed at the La locus in the La-knockout clones used in this study (La-ko3 through La-ko5). Analysis performed with CRISPResso2^47^. **f**, Cumulative population doublings of parental, La-ko4, and La-ko5 K562 PEmax cells. **g**, Flow cytometry analysis of GFP expressed from the PEmax construct at the AAVS1 locus in K562 PEmax parental, La-ko3, La-ko4, and La-ko5 cells. Data collected from cells prior to transfection for experiment depicted in Fig. 2c. **h**, Percentages of prime editing (PE3) outcomes across ten edits with pegRNAs (top) or epegRNAs (bottom) at five genomic loci in HEK293T cells with and without depletion of La by siRNA. Fold-changes in outcome frequencies presented in Fig. 2e. Editing components delivered by plasmid transfection in a, b and h. Data and error bars in a, b and h indicate mean ± s.d. (n = 4 independent biological replicates). Data in d, e and g depict results from characterizations of n = 1 cell lines. Percentages in f indicate relative mean ± s.d. (n = 3 independent biological replicates measured across an 8-day time course) of daily fold changes in cell numbers, essentially the relative percentages of cells to expect after one day of growth for La-ko4 and La-ko5 compared with parental K562 PEmax cells. *P*-values in h are from one-tailed unpaired Student’s *t*-test.

La, a ubiquitously expressed eukaryotic protein, is involved in diverse aspects of RNA metabolism, but one of its most well characterized roles is binding polyuridine (polyU) tracts at the 3′ ends of nascent RNA polymerase III (Pol III) transcripts and protecting them from exonucleases^2,17^. Because our genome-scale CRISPRi screens relied on a Pol III-transcribed pegRNA, the *La* phenotypes we observed from those screens may represent an interaction between La and that pegRNA. Before evaluating this possibility, we used our reporter system and two *La*-targeting CRISPRi sgRNAs, each of which depleted *La* mRNA by >89% (Fig. 1d), to validate the effect of La on prime editing. We made three observations. (1) Loss of La consistently impaired intended editing, with defects observed across approaches (PE2, PE3, PE4 and PE5), two different edits (+7 GG-to-CA substitution and +1 21-bp His-tag insertion) and when using either pegRNAs or an epegRNA^18^ (Fig. 1e and Extended Data Fig. 3a,b); however, the effect was substantially weaker with the epegRNA. (2) Defects were observed when MMR was suppressed (PE4 and PE5)^5^ and when installing an edit that should evade MMR owing to its length (21-bp insertion)^19^. (3) Loss of La reduced the frequencies of intended edits with and without accompanying insertions or deletions (indels) but not outcomes with indels alone (Fig. 1e). These results show that the role of La in prime editing is orthogonal to MMR and primarily affects installation of the intended edit.

**Fig. 2:**
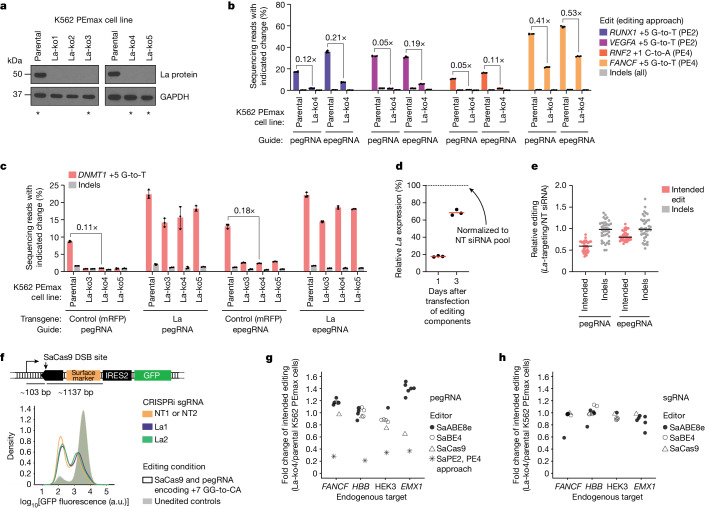
La promotes prime editing across edit types and genomic loci. **a**, Western blot analysis of K562 cells constitutively expressing PEmax (K562 PEmax parental) and clones with genetic disruption of *La* (La-ko1 through La-ko5). Asterisks indicate cell lines used in this study. See also Extended Data Fig. 3d. **b**, Percentages of prime editing outcomes at indicated genomic loci. pegRNAs and epegRNAs (evopreQ_1_) were delivered as plasmids without or with MLH1dn (PE2 or PE4, respectively). **c**, Percentages of prime editing outcomes with or without ectopic expression of *La*. Expression plasmids for La or mRFP control were delivered alongside plasmids encoding pegRNA or epegRNA (evopreQ_1_). The PE2 approach was used. **d**, Quantification of RNAi-mediated *La* depletion. RT–qPCR from HEK293T cells. Data normalized to *ACTB* and presented relative to the non-targeting (NT) siRNA pool. **e**, Fold changes in prime editing outcomes across ten PE3 edits (substitutions, insertions and deletions) at five genomic loci in HEK293T cells with or without *La* depletion. Editing percentages are presented in Extended Data Fig. 3h. **f**, Top, schematic of the MCS reporter, including distances between the predicted SaCas9 cut site and sequences required for GFP expression. Bottom, flow cytometry analysis of MCS reporter cells with and without CRISPRi-mediated *La* depletion after induction of SaCas9-mediated DSB and unedited controls. Quantification presented in Extended Data Fig. 4a. **g**,**h**, Fold changes in editing outcomes induced with pegRNA (**g**) or sgRNA (**h**) using SaABE8e, SaBE4, SaCas9 or SaPE2 (PE4 approach, **g** only) in La-ko4 relative to parental K562 PEmax cells (intended edits only). Editing percentages presented in Extended Data Fig. 4c–f. Editing components were delivered by plasmid transfection in **b**,**c** and **e**–**h**. Data and error bars in **b** and **c** indicate the mean ± s.d. (*n* = 4 and 3 independent biological replicates, respectively). Horizontal bars in **d** and **e** indicate geometric means (*n* = 3 independent biological replicates) and medians of fold changes (10 edits, each with *n* = 4 independent biological replicates plotted individually), respectively. Data in **g** and **h** represent ratios of means for individual editing outcomes (*n* = 3 independent biological replicates for each outcome).

We next tested the impact of La on prime editing at several endogenous loci using an optimized SpCas9-based prime editor: PEmax^5^. For these experiments, we engineered a K562 cell line that constitutively expresses PEmax from the AAVS1 safe-harbour locus^20^ (K562 PEmax parental cells) and derived *La* knockout clones (La-ko1–La-ko5) (Fig. 2a and Extended Data Fig. 3c–e). Consistent with experiments using our reporter system, intended editing efficiencies were reduced in *La* knockout cells compared with parental K562 PEmax cells using either pegRNAs or epegRNAs with the PE2 or PE4 approach (with a weaker effect again observed for epegRNAs) (Fig. 2b,c). Additionally, ectopic expression of *La* rescued intended editing (Fig. 2c), and no obvious relationship was observed between editing efficiencies and cell growth or PEmax expression in the *La* knockout lines (Extended Data Fig. 3f,g).

To determine whether the role of La in prime editing is cell-type or edit-type specific, we evaluated PE3 in HEK293T cells transfected with *La*-targeting or non-targeting small interfering RNAs (siRNAs) (Fig. 2d,e and Extended Data Fig. 3h). Sequencing of five genomic loci, each targeted with a substitution and an insertion or deletion edit, revealed decreased intended editing efficiencies in *La*-depleted cells, with a median reduction of 39.7% for pegRNAs and 19.2% for epegRNAs. Phenotypes from this experiment were generally weaker than those observed with *La* knockout cells, probably due to the rebound of *La* expression from RNAi-mediated depletion during the experiment (Fig. 2d). Alongside the observation that ectopic expression of *La* increased intended editing in parental cells (2.6-fold and 1.7-fold with pegRNA and epegRNA, respectively) (Fig. 2c), this observation indicates a gene dosage effect.

Throughout these experiments, we tested both pegRNAs and epegRNAs. The latter contain structured motifs at their 3′ ends and can enhance prime editing, with improvements loosely attributed to pegRNA stabilization^18^. Loss of La decreased editing with both pegRNAs and epegRNAs, but phenotypes were consistently stronger with pegRNAs (Fig. 2b,c,e and Extended Data Fig. 3a,b,h). This difference fits a model wherein La promotes editing by interacting with the 3′ ends of pegRNAs and epegRNAs but has a stronger effect on pegRNAs, of which the less structured 3′ ends may be less stable or more accessible to La.

## Loss of La does not consistently affect other editing modalities

**Extended Data Fig. 4:**
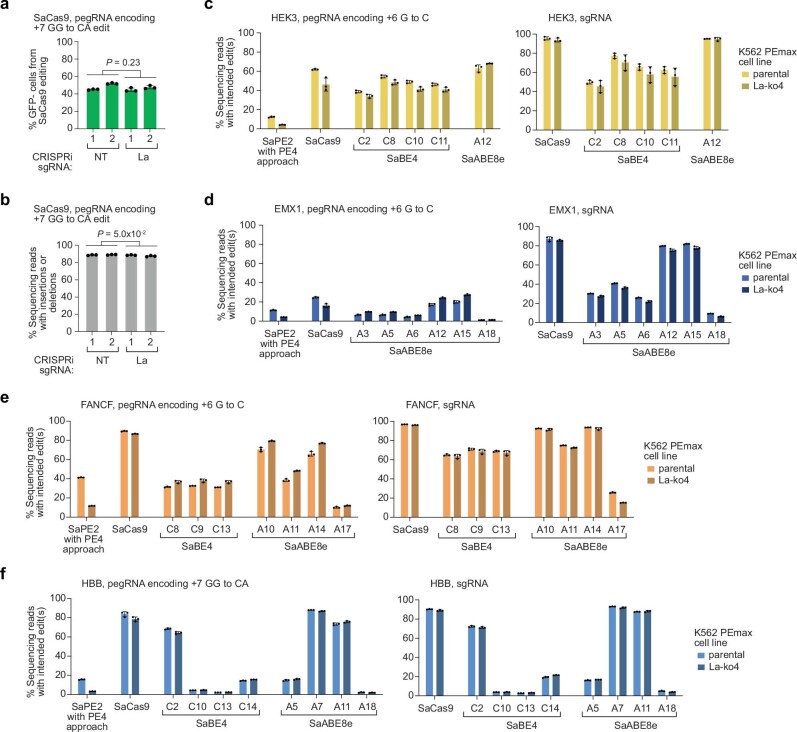
La has a stronger impact on prime editing than other editing modalities. **a**, Percentages of GFP- cells within indicated cell populations arising from SaCas9-induced DSBs at a stably integrated MCS reporter in K562 CRISPRi cells. CRISPRi sgRNAs were delivered by lentiviral transduction. Editing components (SaCas9, +7 GG to CA pegRNA) were delivered by plasmid transfection. Representative flow cytometry data from each condition and unedited controls also presented in Fig. 2f. **b**, Quantification of SaCas9-induced indels at stably integrated MCS reporter described in a. **c-f**, Percentages of intended editing achieved in K562 PEmax parental and La*-*ko4 cells using SaPE2 with the PE4 approach, SaCas9, SaBE4, and SaABE8e across four genomic loci, HEK3 (c), EMX1 (d), FANCF (e) and HBB (f). The same pegRNA or sgRNA expression plasmid was used for all editing systems at each target, with select combinations excluded (SaPE2 with PE4 approach with any sgRNA and SaBE4 at EMX1). Relative editing for each intended outcome presented in Fig. 2g and h. Data and error bars in a-f indicate mean ± s.d. (n = 3 independent biological replicates). *P*-values in a and b are from two-tailed unpaired Student’s *t*-test.

Prime editing relies on pegRNA 3′ extensions, whereas other Cas9-based genome-editing modalities do not. To test whether loss of La impairs Cas9-mediated gene disruption, we examined editing at the MCS reporter target site in our MCS reporter cells using SaCas9^21^ and the +7 GG-to-CA pegRNA (Fig. 2f). The MCS reporter target site is positioned 103 bp downstream and 1,137 bp upstream of a promoter and an IRES required for GFP expression, respectively, and is thus within an approximately 1.2-kb region that does not contain any sequence required for expression of that marker gene. Nevertheless, consistent with previous observations that Cas9-induced DNA double-strand breaks (DSBs) can generate large deletions and disrupt genes distant from the target site^10,22^, editing at this target caused loss of GFP. Neither GFP loss nor the frequencies of small, DSB-induced indels at the target site, however, were significantly altered by *La* depletion (Fig. 2f and Extended Data Fig. 4a,b), which suggested that La had no effect on either type of outcome. We next selected four genomic targets at which four corresponding pegRNAs were able to elicit editing with SaCas9, two base editing systems (SaBE4-Gam^23^ and SaABE8e^24^) and SaPE2 using the PE4 approach. We then transfected plasmids encoding each of these four pegRNAs or sgRNAs with the same spacers (with other editing components) into our K562 PEmax parental and La-ko4 cells. Amplicon sequencing revealed that loss of La had the strongest and most consistent effect on prime editing and moderate or inconsistent effects on other approaches using pegRNAs, with minimal effects when editing with sgRNAs (Fig. 2g,h and Extended Data Fig. 4c–f). We therefore conclude that La has a specific effect on prime editing, which may arise from a specialized role in prime editing (for example, 3′ extension stability) or from promoting processes generally required by Cas9-based technologies but to which prime editing may be more sensitive (for example, effector complex formation or level).

## La interacts with and stabilizes 3′ ends of polyuridylated pegRNAs

**Fig. 3:**
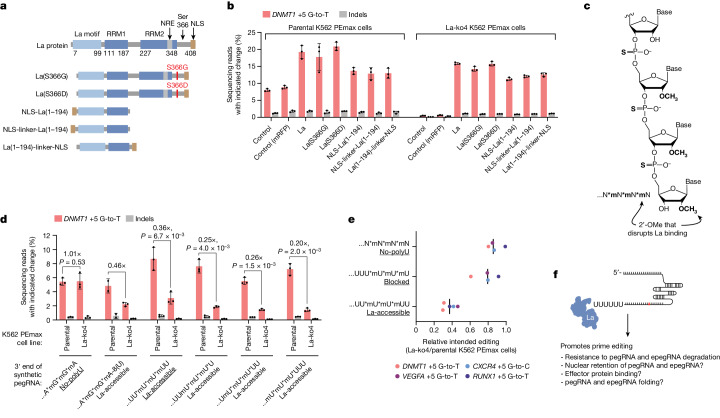
La functionally interacts with the 3′ ends of polyuridylated pegRNAs. **a**, Domain architectures of La and mutants. NRE, nuclear retention element Linker, SGGS. **b**, Percentages of prime editing outcomes with or without ectopic expression of *La* or mutants depicted in **a**. Expression plasmids were delivered to indicated cells alongside the plasmid encoding the *DNMT1* +5 G-to-T pegRNA. **c**, Schematic of RNA with chemical modifications (bold); specifically, phosphorothioate bonds (asterisks in sequence representation) and 2′-OMe modifications (‘m’ in sequence representation). **d**, Percentages of prime editing outcomes produced using 100 pmole of synthetic pegRNAs with indicated 3′ end configurations. **e**, Fold changes in average intended prime editing at four genomic loci in La-ko4 cells relative to parental K562 PEmax cells produced using 100 pmole of synthetic pegRNA with the indicated 3′ end configurations. Editing percentages provided in Extended Data Fig. 5e. **f**, Model of La interaction with pegRNAs. The PE2 approach was used in **b**,**d**,**e**. Underlining in **d**,**e** indicates particular 3′ end configuration patterns. Data and error bars in **b** and **d** indicate the mean ± s.d. (*n* = 2–3 independent biological replicates). Vertical bars in **e** indicate medians (4 edits) of ratios of means (*n* = 3 independent biological replicates for each edit). *P* values in **d** are from one-tailed unpaired Student’s *t*-test. Image of pegRNA in **f** adapted from ref. ^40^, Elsevier.

La is a 408-residue protein that consists of a highly conserved La motif, two RNA recognition motifs (RRM1 and RRM2) and a flexible region with a nuclear localization signal (NLS) at the C terminus^25^ (Fig. 3a). The N-terminal domain of La (La(1–194)), which contains the La motif and RRM1, is necessary and sufficient for high-affinity binding to 3′ polyU^25,26^, whereas phosphorylation of Ser366 at the C terminus has been implicated in transcriptional modulation through Pol III recycling^27^. We reasoned that if La promotes prime editing through transcription, truncation of the C-terminal domain or mutation of Ser366 could substantially alter its effects, but if La promotes prime editing by binding to the 3′ polyU of pegRNAs, La(1–194) should be sufficient to promote prime editing. To test this idea, we evaluated prime editing in K562 PEmax parental and La-ko4 cells transfected with *La* or *La* mutants (Fig. 3a). The results showed that expression of full-length La, two Ser366 mutants (S366D and S366G)^27^ or La(1–194) fused to a NLS in different configurations all rescued prime editing in *La* knockout cells. Moreover, each La(1–194) construct was sufficient to rescue editing to levels higher than those observed in parental cells without ectopic *La* or mutant expression, but Ser366 mutants and full-length La were moderately more potent than La(1–194) constructs (Fig. 3b). These results establish that La promotes prime editing primarily through the N-terminal domain, with contribution from the C terminus, but little to no contribution from Ser366.

**Extended Data Fig. 5:**
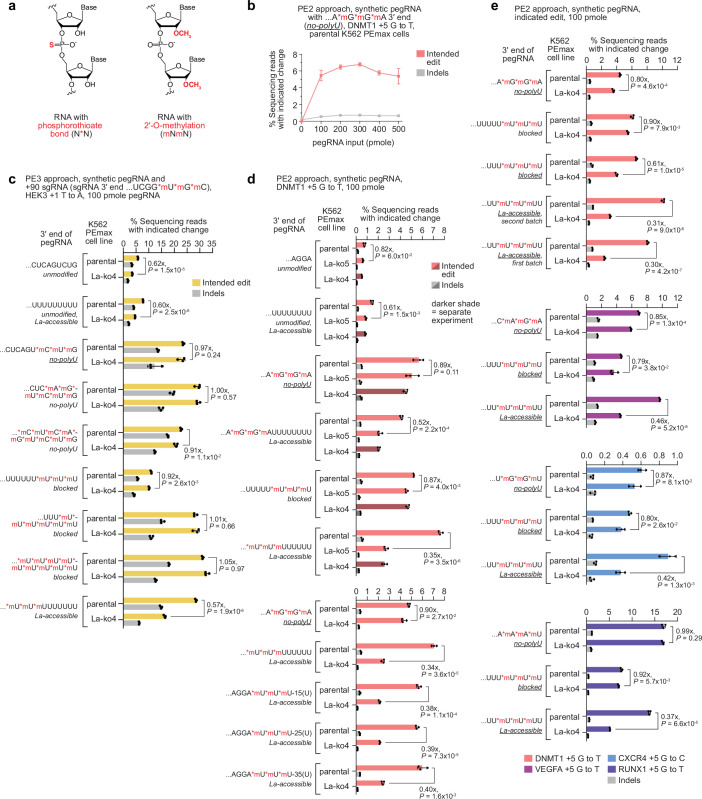
Prime editing with synthetic pegRNAs designed to block or allow La binding reveals functional interaction between La and polyuridylated 3′ ends. **a**, Chemical structures of ribonucleotides linked by a phosphorothioate bond (left) or with substitution of ribose 2′-OH for 2′-O-methyl groups (2′-OCH_3_) (right). **b**, Percentages of prime editing outcomes at the endogenous DNMT1 locus in parental K562 PEmax cells using one synthetic pegRNA with the indicated 3′ end configuration. Input was titrated from 0 to 500 pmole. **c**, **d**, Percentages of prime editing outcomes at the endogenous HEK3 (c) and DNMT1 (d) loci in K562 PEmax cells using 100 pmole of synthetic pegRNAs and 50 pmole of synthetic sgRNA (c only) with specified 3′ end sequences and chemical modifications. **e**, Percentages of prime editing outcomes at endogenous DNMT1, CXCR4, VEGFA, and RUNX1 loci in K562 PEmax parental and La-ko4 cells using 100 pmole of synthetic pegRNAs with indicated 3′ end configurations. Fold-changes in outcome frequencies also presented in Fig. 3e. Data and error bars in b-e indicate mean ± s.d. (n = 3 independent biological replicates). *P*-values in c-e are from one-tailed unpaired Student’s *t*-test.

To determine whether the role of La in prime editing is contingent on an ability to bind pegRNA 3′ polyU, we designed and tested synthetic pegRNAs with or without 3′ polyU and different patterns of 3′ chemical modifications, including 2′-O-methylation (2′-OMe; indicated as ‘m’ in sequence representations) and phosphorothioate linkages (indicated as asterisks in sequence representations) (Extended Data Fig. 5a–d). Three considerations guided the design of these pegRNAs. (1) Chemical modifications, including 2′-OMe and phosphorothioate linkages, confer resistance to RNA exonucleases and are therefore often included at the ends of synthetic guide RNAs to improve editing efficiencies^28^. We observed that pegRNAs with various patterns of 3′ chemical modifications (no-polyU, blocked or La-accessible) produced higher intended prime editing efficiencies in K562 PEmax parental cells than those without (unmodified or unmodified, La-accessible), which confirmed the benefit of such modifications (Extended Data Fig. 5c,d). (2) La(1–194) can bind polyU at the 3′ ends of RNA with nanomolar affinity in vitro, but substituting uridines within the polyU for other nucleotides reduces binding affinity with varying degrees (1.4-fold to 14-fold)^26^. Therefore, the addition of polyU to the 3′ ends of pegRNAs should promote interactions with La. We observed that adding terminal uridines to pegRNAs with otherwise unmodified 3′ ends increased intended editing efficiencies in K562 PEmax parental cells (unmodified, La-accessible versus unmodified). However, improvements were minimal, especially compared with enhancement from chemically modifying the 3′ ends. (3) Replacing the ribose 2′-hydroxyl group (2′-OH) of the most terminal uridine of an RNA oligomer with 2′-OMe strongly disrupts La(1–194) binding to 3′ polyU (38-fold reduction of binding affinity in vitro), presumably by creating a steric block^26^ (Fig. 3c). We observed that pegRNAs with a terminal 2′-OMe and with or without a polyU tail (blocked and no-polyU, respectively) were minimally or not affected by La loss. By contrast, those with chemical modifications near their 3′ ends but upstream of unmodified polyU tails (La-accessible) were compromised for intended editing in La-ko4 cells. We next tested synthetic pegRNAs with additional 3′ end configurations, which confirmed that La strongly affected intended prime editing efficiencies when the last 2′-OH of an appended polyU is kept unmodified (Fig. 3c,d). Moreover, editing four genomic loci with pegRNAs terminating in a La-accessible end (UU*mU*mU*mUU), a blocked end (UUU*mU*mU*mU) or no-polyU ends (N*mN*mN*mN) further supported this conclusion (Fig. 3e and Extended Data Fig. 5e). These results establish an association between the expected capability of pegRNAs to bind La and their reliance on La for editing and demonstrate that La can affect prime editing independently of transcription (Fig. 3f).

**Extended Data Fig. 6:**
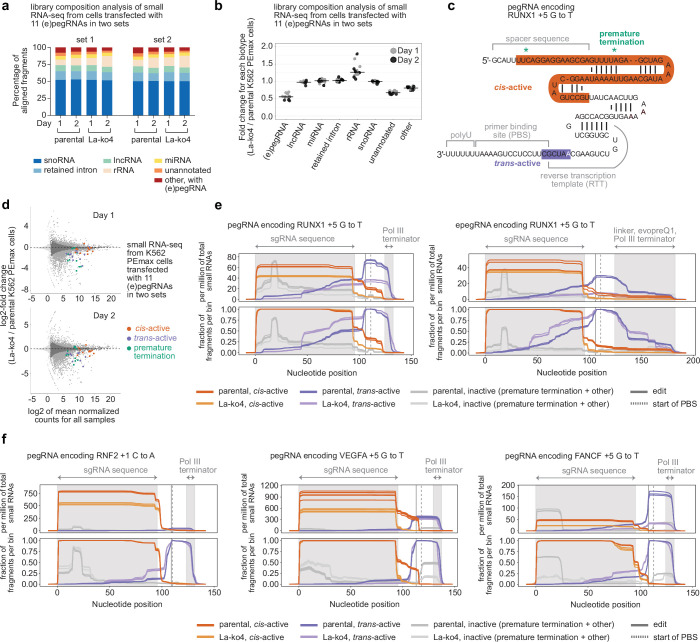
Details of small RNA-seq experiment performed with two sets of (e)pegRNAs. **a**, Composition of small RNA-seq libraries from K562 PEmax parental or La-ko4 cells. Data are from samples collected one and two days after transfection of eleven (e)pegRNAs in two sets. **b**, Fold changes in normalized counts of indicated biotypes in La-ko4 cells relative to parental K562 PEmax cells, from samples collected one and two days after transfection of eleven (e)pegRNAs in two sets. Counts were calculated per replicate for each set of (e)pegRNAs as the sums of properly aligned fragments classified as each biotype and normalized by total RNA counts. **c**, Schematic of minimum sequence defining each class of (e)pegRNA fragments from small RNA-seq (orange*, cis*-active; purple, *trans*-active). Representative sequence used (*i.e*., RUNX1 + 5 G to T pegRNA). Edit-encoding nucleotide (white base) and cryptic terminators (green asterisks) indicated. **d**, Plot (MA) of small RNA-seq data displaying mean normalized expression versus log_2_-fold change in expression of human genes and (e)pegRNA bins from La-ko4 cells relative to parental K562 PEmax cells. Data are from samples collected one (top) and two (bottom) days after transfection of plasmids encoding seven pegRNAs and four epegRNAs. Alignment categories are indicated (gray, human small RNA; orange, *cis*-active; purple, *trans*-active; green, premature termination) and genes with adjusted *p*-values ≤ 0.05 are highlighted in light gray. **e**, Coverage plots of small RNA-seq fragments for the pegRNA (left) or epegRNA (right) specifying RUNX1 + 5 G to T from specified cell lines collected one day after (e)pegRNA plasmid transfection. Data are normalized by counts of fragments from total human small RNA (top) or those within the corresponding bins: *cis*-active, *trans*-active, inactive (bottom). Nucleotide position 0 denotes the 5′ end of the RNA, and positions of the edit-encoding nucleotide (vertical solid line) and the start of PBS (vertical dashed line) are indicated. Shaded areas represent sgRNA sequence and Pol III terminator (pegRNA) or sgRNA sequence, linker, evopreQ_1_, and Pol III terminator (epegRNA). **f**, Coverage plots of small RNA-seq fragments for pegRNAs specifying RNF2 + 1 C to A (left), VEGFA + 5 G to T (middle) or FANCF + 5 G to T (right) from specified cell lines collected one day after (e)pegRNA plasmid transfection. Data are normalized by counts of fragments from total human small RNA (top) or those within the corresponding bins: *cis*-active, *trans*-active, inactive (bottom). Nucleotide position 0 denotes the 5′ end of the RNA, and positions of the edit-encoding nucleotide (vertical solid line) and the start of PBS (vertical dashed line) are indicated. Shaded areas represent sgRNA sequence and Pol III terminator. Data in a indicate means (n = 3 independent biological replicates). Horizontal bars in b indicate medians (12 data points per biotype, each biotype has n = 3 independent biological replicates for each day and each set of (e)pegRNAs). Data in d were calculated from n = 6 (VEGFA + 5 G to T) and 3 (all others) independent biological replicates and adjusted *P*-values were calculated by DESeq2^33^ using the two-tailed Wald test with Bonferroni-Holm correction. Coverages in e and f represent n = 6 (VEGFA + 5 G to T) and 3 (all others) independent biological replicates. Image of pegRNA in c adapted from ref. ^64^, Springer Nature America.

**Extended Data Fig. 7:**
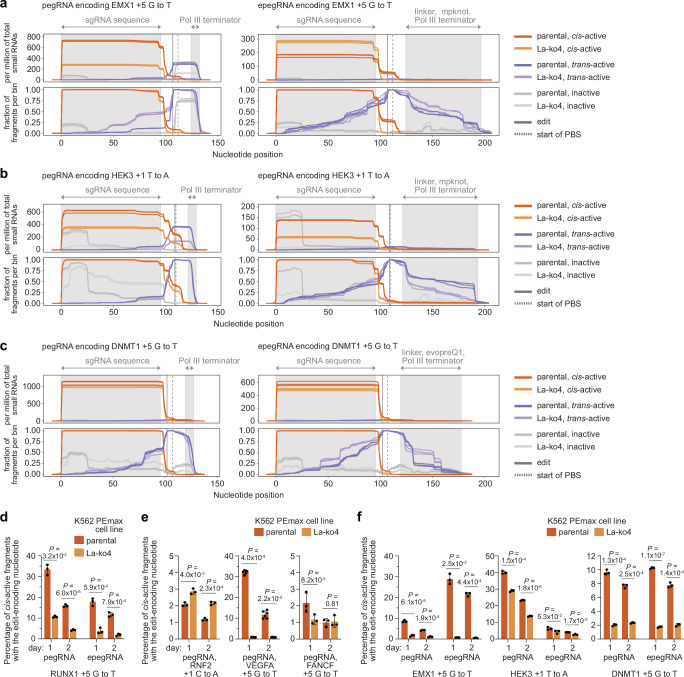
Additional details of small RNA-seq experiment performed with two sets of (e)pegRNAs. **a-c**, Coverage plots of small RNA-seq fragments for pegRNAs (left) or epegRNAs (right) encoding EMX1 + 5 G to T (a), HEK3 + 1 T to A (b) or DNMT1 + 5 G to T (c) from specified cell lines collected one day after (e)pegRNA plasmid transfection. Data are normalized by counts of fragments from total human small RNA (top) or those within the corresponding bins: *cis*-active, *trans*-active, inactive (bottom). For representative schematic of bins, see Extended Data Fig. 6c. Nucleotide position 0 denotes the 5′ end of the RNA, and positions of the edit-encoding nucleotide (vertical solid line) and the start of PBS (vertical dashed line) are indicated. Shaded areas represent sgRNA sequence, and Pol III terminator for pegRNAs and linker plus evopreQ_1_/mpknot and Pol III terminator for epegRNAs. **d**, Percentages of *cis*-active fragments with the edit-encoding nucleotide for the pegRNA (left) and the epegRNA (right) specifying RUNX1 + 5 G to T in K562 PEmax parental or La-ko4 cells. Associated coverage plots presented in Extended Data Fig. 6e. **e**, Same as d but for pegRNAs specifying RNF2 + 1 C to A (left), VEGFA + 5 G to T (middle) or FANCF + 5 G to T (right). Associated coverage plots presented in Extended Data Fig. 6f. **f**, Same as d but for pegRNAs and epegRNAs specifying EMX1 + 5 G to T (left), HEK3 + 1 T to A (middle) or DNMT1 + 5 G to T (right). Associated coverage plots presented in a-c. Coverages depicted in a-c represent n = 3 independent biological replicates. Data and error bars in d-f indicate mean ± s.d. (n = 6 and 3 independent biological replicates for VEGFA + 5 G to T and all others, respectively). *P*-values in d-f are from two-tailed unpaired Student’s *t*-test.

**Extended Data Fig. 8:**
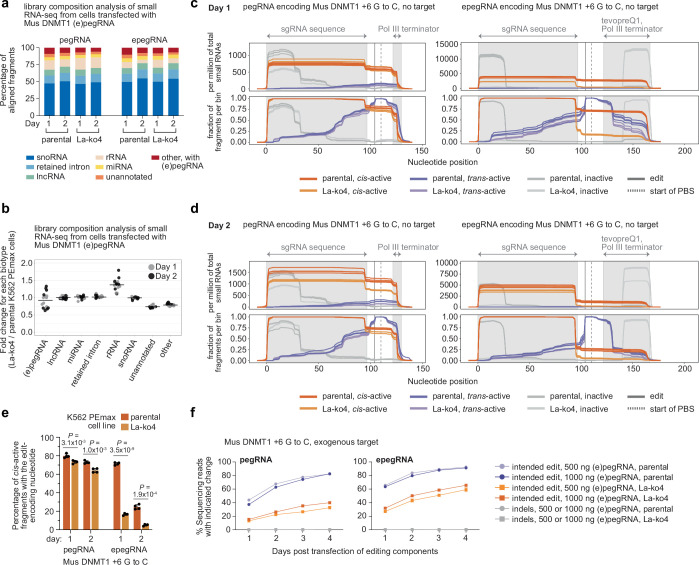
Details of small RNA-seq experiment performed with non-targeting pegRNA and epegRNA, each specifying a + 6 G to C edit in a target site adapted from the *Mus musculus* DNMT1 gene. **a**, Composition of small RNA-seq libraries from K562 PEmax parental or La-ko4 cells. Data from samples collected one and two days after transfection of plasmid encoding a pegRNA or an epegRNA specifying mouse DNMT1 + 6 G to C. **b**. Fold changes in normalized counts of indicated biotypes in La-ko4 cells relative to parental K562 PEmax cells, from samples collected one and two days after transfection of plasmid encoding a pegRNA or an epegRNA specifying mouse DNMT1 + 6 G to C. Counts were calculated per replicate for the pegRNA and the epegRNA as the sums of properly aligned fragments classified as each biotype and normalized by total RNA counts. **c**, **d**, Coverage plots of small RNA-seq fragments for the pegRNA (left) or the epegRNA (right) specifying mouse DNMT1 + 6 G to C edit from specified cell lines, which lack the (e)pegRNA target, collected one (c) and two (d) days after (e)pegRNA plasmid transfection. Data are normalized by counts of fragments from total human small RNA (top) or those within the corresponding bins: *cis*-active, *trans*-active, inactive (bottom). Nucleotide position 0 denotes the 5′ end of the RNA, and positions of the edit-encoding nucleotide (vertical solid line) and the start of PBS (vertical dashed line) are indicated. Shaded areas represent sgRNA sequence, and Pol III terminator for the pegRNA and tevopreQ_1_ plus Pol III terminator for the epegRNA. **e**, Percentages of *cis*-active fragments with the edit-encoding nucleotide for the pegRNA (left) and the epegRNA (right) specifying mouse DNMT1 + 6 G to C edit in K562 PEmax parental or La-ko4 cells without the (e)pegRNA target. Associated coverage plots presented in c and d. **f**, Percentages of prime editing outcomes in K562 PEmax parental and La-ko4 cells transduced with the mouse DNMT1 target and transfected with either the pegRNA or epegRNA plasmid specifying mouse DNMT1 + 6 G to C. Data are from samples collected on indicated days. Data in a indicate means (n = 4 independent biological replicates). Horizontal bars in b indicate medians (16 data points per biotype, each biotype has n = 4 independent biological replicates for the pegRNA and epegRNA on each day). Coverages depicted in c and d represent n = 4 independent biological replicates. Data and error bars in e and f indicate mean ± s.d. (n = 4 and 3 independent biological replicates, respectively). *P*-values in e are from two-tailed unpaired Student’s *t*-test.

Although several possible mechanisms could explain how an interaction between La and pegRNA 3′ polyU could promote prime editing (Fig. 3f), recent studies have shown that pegRNA 3′ ends are degraded within cells^18,29–31^ and that truncated pegRNAs can interfere with prime editing^18^. We therefore used small RNA sequencing to explore the possibility that La affects the stability and integrity of pegRNAs and epegRNAs (Extended Data Figs. 6–8). Loss of La destabilized Pol III-transcribed pegRNAs and epegRNAs and rendered their 3′ ends particularly unstable. However, careful consideration of those effects (Supplementary Discussion) suggested that their relationship to editing efficiency may be complex (nonlinear) and/or that protecting pegRNAs and epegRNAs may represent only part of the role that La has in prime editing (Fig. 3f). These data nevertheless provide further support for a functional interaction between La and the 3′ ends of polyuridylated pegRNAs.

## The PE7 editor enhances prime editing

**Fig. 4:**
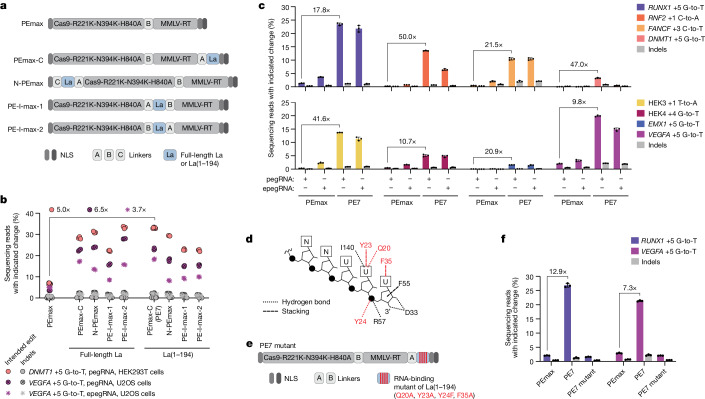
Fusion of the La RNA-binding, N-terminal domain to PEmax improves prime editing. **a**, Schematics of prime editor architectures. Medium grey NLS, bipartite NLS (SV40); dark grey NLS, NLS (c-Myc); A, B, C, linkers (Methods); MMLV-RT, human codon-optimized MMLV-RT. **b**, Percentages of prime editing outcomes produced with editors from **a**, pegRNAs or an epegRNA (evopreQ_1_), and the PE2 approach at *DNMT1* and *VEGFA* loci in indicated cells. **c**, Percentages of prime editing outcomes at eight endogenous loci in U2OS cells using pegRNAs or epegRNAs (HEK3, mpknot; HEK4, tevopreQ_1_; all other loci, evopreQ_1_) and the PE2 approach. Data from pegRNAs also plotted in Extended Data Fig. 11a. **d**, Schematic of interactions between the La N-terminal domain and RNA with 3′-UUU_OH_^26^. Red font and red lines indicate residues mutated in the PE7 mutant (Q20, Y23, Y24 and F35) and associated interactions. **e**, Schematic of the PE7 mutant harbouring four mutations (red font and red vertical lines) in La(1–194) to disrupt 3′ polyU binding. **f**, Percentages of prime editing outcomes produced with PEmax, PE7 or the PE7 mutant at *RUNX1* and *VEGFA* loci in U2OS cells with the PE2 approach and pegRNAs. Editing components were delivered by plasmid transfection in **b**,**c**,**f**. Data in **b** indicate values of independent biological replicates (*n* = 9 for PEmax and *n* = 6 for PE7 with *DNMT1* edit; *n* = 4 for PEmax with *VEGFA* edit; *n* = 3 for all others). Data and error bars in **c** and **f** indicate the mean ± s.d. (*n* = 3 independent biological replicates).

Given the evidence showing that La promotes prime editing primarily through La(1–194), we next asked whether tethering that domain to a prime editor protein could offer improvement. Fusing full-length La or La(1–194) to PEmax in multiple positions (that is, the N terminus, the C terminus or between Cas9 nickase and MMLV-RT) improved intended editing efficiencies in U2OS and HEK293T cells when evaluated with the PE2 approach using transiently expressed pegRNAs and one epegRNA (Fig. 4a,b). Among the constructs with full-length La, the highest median intended editing was achieved with an internal fusion (PE-I-max-2) and, among La(1–194) fusion constructs, a C-terminal fusion (PEmax-C) was the most efficient. We named the latter PE7.

**Extended Data Fig. 9:**
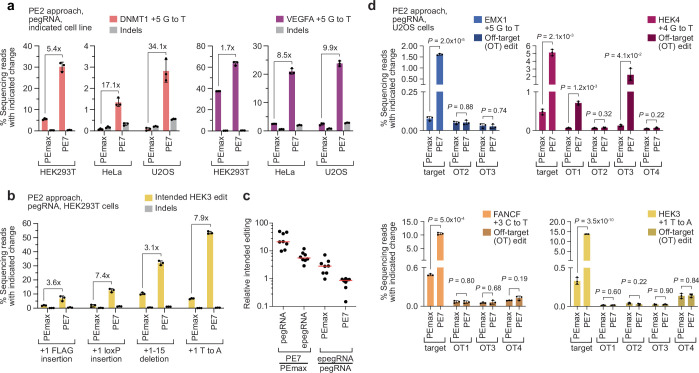
PE7 enhances prime editing in different cell lines and with different edit types with minimal effect on off-target editing. **a**, Percentages of prime editing outcomes at DNMT1 and VEGFA loci in HEK293T, HeLa, and U2OS cells. **b**, Percentages of prime editing outcomes at HEK3 locus in HEK293T cells. **c**, Fold changes in intended prime editing. Editing percentages in Fig. 4c. **d**, Percentages of editing outcomes produced by PEmax or PE7 with the PE2 approach at on- and off-target sites using pegRNAs targeting the EMX1 (top left), HEK4 (top right), FANCF (bottom left), and HEK3 (bottom right) loci in U2OS cells. On-target editing data also presented in Fig. 4c and Extended Data Fig. 11a. Editing components delivered by plasmid transfection in a-d. Data and error bars in a, b and d indicate mean ± s.d. (n = 3 independent biological replicates). Horizontal bars in c indicate medians (8 edits) of ratios of means (n = 3 independent biological replicates for each edit). *P*-values in d are from two-tailed unpaired Student’s *t*-test.

Subsequent characterization of PE7 revealed substantial improvement compared with PEmax across eight genomic loci, three cell lines (HEK293T, HeLa and U2OS) and distinct edit types (single-nucleotide substitutions, insertions or a 15-bp deletion), with the largest improvements observed in MMR-proficient HeLa and U2OS cells (Fig. 4c and Extended Data Fig. 9a–c). In particular, PE7 improved intended editing efficiencies in U2OS cells with the PE2 approach by 21.2-fold and 5.5-fold (median) using transiently expressed pegRNAs and epegRNAs, respectively, while maintaining low frequencies of on-target indels (Fig. 4c and Extended Data Fig. 9c). Additionally, PE7 had minimal impact on off-target editing compared with PEmax, significantly increasing editing frequencies at only 2 of 13 off-target loci examined^4,5,18,32^ (Extended Data Fig. 9d and Supplementary Discussion). Results from U2OS cells also showed that, despite increasing baseline editing with PEmax, epegRNAs gave no additional improvement relative to pegRNAs when using PE7 (Fig. 4c and Extended Data Fig. 9c). Instead, pairing PE7 with epegRNAs produced intended editing efficiencies that were similar to or lower than those from PE7 and pegRNAs. Reduced affinity towards Cas9^18^, differences in expression^18^ or compromised binding to La(1–194) may explain the relatively worse performance of epegRNAs with PE7. Alternatively, if PE7 and epegRNAs improve prime editing through similar mechanisms, PE7 may have a dominant effect.

**Extended Data Fig. 10:**
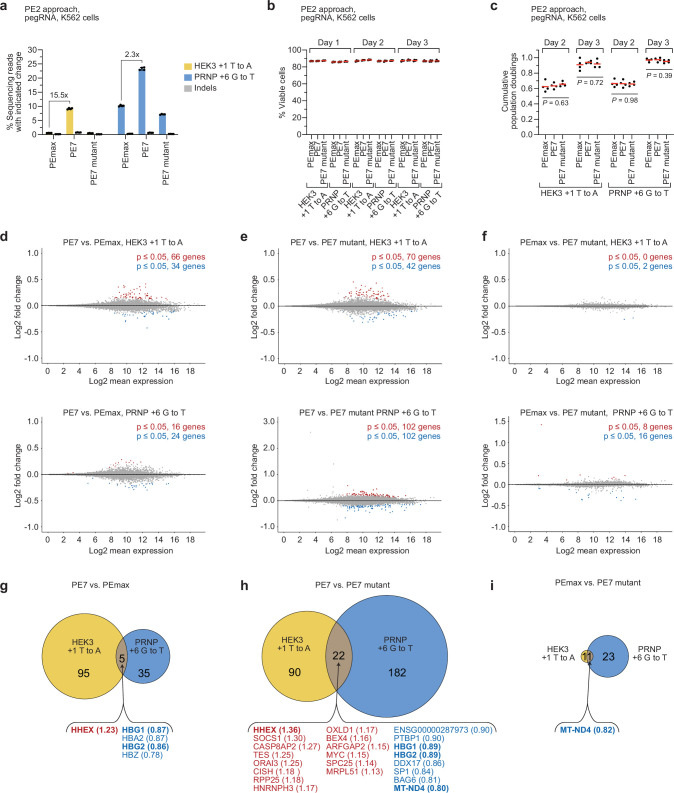
PE7 has negligible effects on cell viability, cell growth, and mRNA abundance compared with PEmax and PE7 mutant. **a**, Percentages of prime editing outcomes at the endogenous HEK3 and PRNP loci in K562 cells using PEmax, PE7 or PE7 mutant. Editing components delivered by plasmid transfection. Cells from this experiment were also used for analyses in b-i. **b**, Percentages of viable K562 cells quantified by flow cytometry one, two, and three days after transfection of pegRNA plasmid specifying either HEK3 + 1 T to A or PRNP + 6 G to T and PEmax, PE7, or PE7 mutant encoding plasmid. **c**, Cumulative population doublings of K562 cells two and three days after transfection of pegRNA plasmid specifying either HEK3 + 1 T to A or PRNP + 6 G to T and PEmax, PE7, or PE7 mutant encoding plasmid. **d-f**, Plot (MA) of RNA-seq data displaying mean normalized gene expression versus log_2_-fold change in gene expression from K562 cells edited with PE7 relative to PEmax (d), PE7 relative to PE7 mutant (e), and PEmax relative to PE7 mutant (f). Analyses were performed with cells edited using two different pegRNAs, one specifying HEK3 + 1 T to A (top) and one specifying PRNP + 6 G to T (bottom). Upregulated and downregulated genes with adjusted *P*-values ≤ 0.05 are highlighted in red and blue, respectively. **g-i**, Venn diagrams of differentially expressed genes (*p* ≤ 0.05) in K562 cells edited at two different loci across three comparisons: PE7 relative to PEmax (g), PE7 relative to PE7 mutant (h), and PEmax relative to PE7 mutant (i). Bolded genes represent those significantly changed in more than one of the indicated comparisons. Data and error bars in a indicate mean ± s.d. (n = 4 independent biological replicates). Horizontal bars in b and c indicate means (n = 4 independent biological replicates). *P*-values in c are from one-way ANOVA. RNA-seq analyses presented in d-i were from n = 4 independent biological replicates. Adjusted *P*-values used for d-i calculated by DESeq2^33^ using the two-tailed Wald test with Benjamini-Hochberg correction.

To confirm that the effect of PE7 on prime editing was due to the RNA-binding activity of the fused La(1–194), we next generated a PE7 mutant with four mutations that have previously been shown to disrupt interactions between La(1–194) and polyuridylated RNA^26^ (Fig. 4d,e). Supporting our model that La promotes prime editing through interactions with pegRNA 3′ ends (Fig. 3f), these mutations abolished improvements from fusing La(1–194) to PEmax when evaluated with four edits in two cell lines (U2OS and K562) (Fig. 4f and Extended Data Fig. 10a).

We next asked whether PE7 causes deleterious effects on cell growth or alters gene expression. Editing with PE7 in K562 cells produced negligible changes to cell viability and caused no significant difference in the number of population doublings observed during editing relative to editing with PEmax and the PE7 mutant (Extended Data Fig. 10b,c). Gene expression analysis^33^ of cells transfected with PEmax, PE7 or the PE7 mutant with *PRNP*-targeting or HEK3-targeting pegRNAs also revealed minimal differences in the cellular transcriptome (mRNA). That is, only one gene was more than twofold upregulated or downregulated significantly in any comparisons made, and only four genes were similarly and significantly changed (Extended Data Fig. 10d–i). We therefore found no evidence of substantial changes to cellular homeostasis.

## Disease-relevant prime editing with PE7

**Fig. 5:**
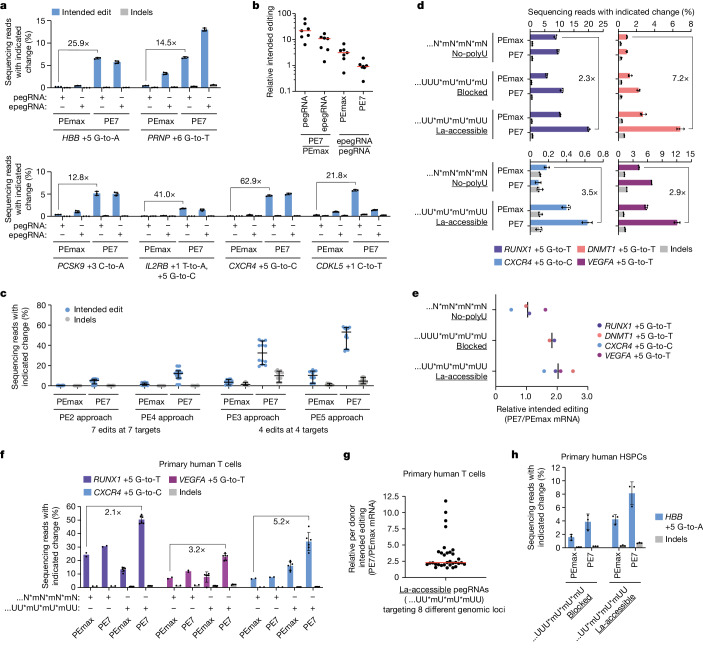
PE7 enhances prime editing at disease-related targets and in primary human cells. **a**, Percentages of prime editing outcomes at six endogenous loci in U2OS cells using pegRNAs and epegRNAs (tevopreQ_1_). Data from pegRNAs also plotted in **c**. **b**, Fold changes in intended prime editing for the six edits in **a** (editing percentages in **a**) and one additional edit for which editing percentages were lower (*HBG1* and *HBG2*). **c**, Prime editing outcome frequencies from indicated approaches (pegRNAs only) in U2OS cells. Data from six endogenous loci in **a** and *HBG1* and *HBG2* (PE2 and PE4) or a subset (PE3 and PE5). **d**, Percentages of prime editing outcomes at four genomic loci in K562 cells using PEmax or PE7 mRNA and synthetic pegRNAs with indicated 3′ end configurations. **e**, Fold changes in average intended prime editing in K562 cells using PE7 mRNA relative to PEmax mRNA for synthetic pegRNAs with indicated 3′ end configurations. Editing percentages in **d**. **f**, Percentages of prime editing outcomes in primary human T cells using PEmax or PE7 mRNA and synthetic pegRNAs with indicated 3′ end configurations. **g**, Fold changes in intended prime editing in primary human T cells using PE7 mRNA relative to PEmax mRNA with La-accessible pegRNAs at eight genomic loci. **h**, Same as **f** but at the *HBB* locus in primary human HSPCs. The PE2 approach was used in **a**,**b**, and **d–h**. Underlining in **d**,**e**,**g**,**h** indicates particular 3′ end configuration patterns. Editing components were delivered by plasmid (**a**–**c**) or RNA (**d**–**h**) transfection. Data and error bars in **a**,**d**,**f**,**h** indicate the mean ± s.d. (*n* = 2–3 independent biological replicates for **a** and **d**; *n* = 6 or 2 donors for **f**; *n* = 3 donors for **h**). Horizontal or vertical bars in **b** and **e** indicate medians (7 and 2/4 edits, respectively) of ratios of means (*n* = 3 independent biological replicates for each edit) and in **c** indicate medians with 99% confidence interval (7 edits for PE2 and PE4, 4 edits for PE3 and PE5, each with *n* = 3 independent biological replicates plotted individually). Data and horizontal bar in **g** indicate ratios of intended editing and median (8 edits, *n* = 4 donors plotted individually).

**Extended Data Fig. 11:**
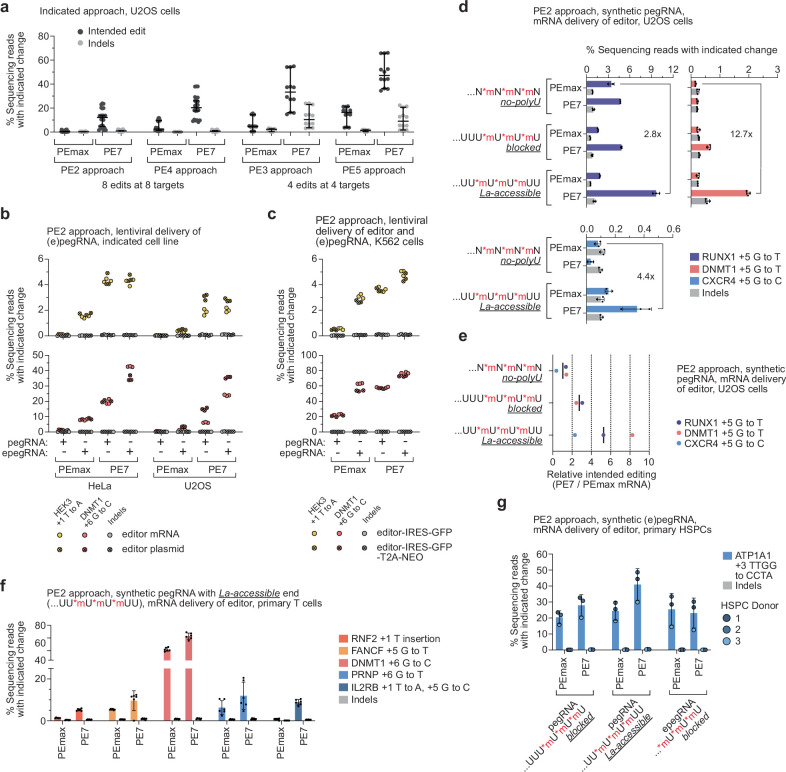
PE7 improves prime editing with different approaches and delivery strategies. **a**, Prime editing outcome frequencies from indicated approaches (pegRNAs only). Data from eight endogenous loci in Fig. 4c (PE2, PE4) or subset (PE3, PE5). **b**, Percentages of prime editing outcomes at endogenous HEK3 (top) and DNMT1 (bottom) loci after transduction of pegRNAs or epegRNAs (tevopreQ_1_) and transfection of PEmax or PE7 editor encoded on mRNA or plasmid in HeLa (left) and U2OS (right) cells. (e)pegRNAs used a modified sgRNA scaffold^65^. **c**, Percentages of prime editing outcomes at endogenous HEK3 (top) and DNMT1 (bottom) loci after transduction of editing components in K562 cells. Two different editor expression constructs (as indicated) were tested. (e)pegRNAs use a modified sgRNA scaffold^65^. epegRNAs use tevopreQ_1_. **d**, Percentages of prime editing outcomes at three genomic loci in U2OS cells using indicated editor mRNA and synthetic pegRNAs with no-polyU, blocked, or La-accessible 3′ end configurations. **e**, Fold changes in average intended prime editing in U2OS cells using PE7 mRNA relative to PEmax mRNA for synthetic pegRNAs with each indicated 3′ end configuration. Editing percentages in d. **f**, Percentages of prime editing outcomes at five genomic loci in primary human T cells using PEmax or PE7 mRNA and synthetic pegRNAs with a La-accessible 3′ end configuration. **g**, Percentages of prime editing outcomes at endogenous ATP1A1 locus in primary human HSPCs using PEmax or PE7 mRNA and synthetic (e)pegRNAs with blocked or La-accessible 3′ end configuration. Editing components delivered as indicted or by plasmid (a) or RNA (d-g) transfection. Data and error bars in d, f and g indicate mean ± s.d. (n = 3 independent biological replicates in d, n = 6 and 3 donors in f and g, respectively). Horizontal bars in a indicate medians with 99% confidence interval (8 edits for PE2/4, 4 edits for PE3/5, each with n = 3 independent biological replicates). Data in b and c indicate individual values of n = 3 independent biological replicates. Vertical bars in e indicate medians (2/3 edits) of ratios of means (n = 3 independent biological replicates for each edit).

We next evaluated editing with PE7 at additional genomic targets^5,18^, including ones associated with sickle cell disease (*HBB*), prion disease (*PRNP*), familial hypercholesterolaemia (*PCSK9*), adoptive T cell transfer therapy (*IL2RB*), HIV infection (*CXCR4*) and CDKL5 deficiency disorder (*CDKL5*) (Fig. 5a,b). Similar to our previous results, editing at these loci with PE7 using the PE2 approach showed substantial improvement over PEmax in U2OS cells (median 21.8-fold and 10.8-fold for pegRNAs and epegRNAs, respectively) (Fig. 5b). Notably, unlike our previous results, we also found one edit (*PRNP* +6 G-to-T) for which use of an epegRNA with PE7 outperformed a matched pegRNA, which indicated that some epegRNAs may synergize with PE7. We then asked whether editing efficiency could be further increased by pairing PE7 with the more efficient PE3, PE4 and PE5 approaches. Across seven disease-relevant edits and our previous set of eight edits (or a subset thereof for PE3 and PE5, which were the only edits tested for those approaches), PE7 produced median 7.3-fold, 7.0-fold and 3.9-fold improvement in intended editing over PEmax, respectively (median 7.2-fold, 7.2-fold and 7.6-fold increases in indels, respectively) (Fig. 5c and Extended Data Fig. 11a). Moreover, when paired with the most advanced system (PE5), PE7 achieved 50.2% median intended editing across eight edits in U2OS cells. PE7 therefore supports substantially increased prime editing efficiency across approaches.

Further evaluating the performance of PE7 with the PE2 approach then revealed that PE7 outperformed PEmax when editors were delivered by plasmids or in vitro transcribed mRNA to HeLa and U2OS cells stably expressing pegRNAs or epegRNAs and when both editors and pegRNAs or epegRNAs were delivered by lentiviral transduction to K562 cells (Extended Data Fig. 11b,c). The latter demonstrated the robustness of PE7 without high-copy delivery. Pairing mRNA-expressed PE7 with La-accessible synthetic pegRNAs (UU*mU*mU*mUU) also produced higher intended editing efficiencies than mRNA-expressed PEmax paired with the same pegRNAs or those with La-blocked (UUU*mU*mU*mU) or no-polyU (N*mN*mN*mN) 3’ end configurations in U2OS and K562 cells (Fig. 5d,e and Extended Data Fig. 11d,e). Moreover, when paired with no-polyU pegRNAs, mRNA-expressed PE7 and PEmax exhibited more comparable performance. These results therefore provide further support for a model wherein an interaction between La and accessible pegRNA 3′ ends promotes prime editing. However, contrary to expectations from experiments in *La* knockout cells (Fig. 3e), PE7 increased intended editing efficiencies relative to PEmax when paired with La-blocked pegRNAs (UUU*mU*mU*mU). This result may be due to enhancement of low-affinity interactions between La(1–194) and La-blocked pegRNAs when in proximity, as in the effector complex or at the site of editing.

Finally, we confirmed that PE7 improves prime editing in primary cells. Consistent with results in K562 and U2OS cells, mRNA-expressed PE7 and La-accessible pegRNAs produced higher intended editing efficiencies than other pairings of mRNA-expressed editors and synthetic pegRNAs in primary human CD3^+^ pan T cells. Overall, 2.1-fold, 3.2-fold and 5.2-fold improvements were achieved compared with more-standard reagents (that is, PEmax with no-polyU pegRNAs) at three different sites (Fig. 5f). Across eight targets in T cells, using mRNA-expressed PE7 with La-accessible pegRNAs achieved a 20.0% median intended editing efficiency with the PE2 approach, which represented a median 2.3-fold improvement compared with PEmax with the same pegRNAs (Fig. 5f,g and Extended Data Fig. 11f). Similarly, prime editing with the PE2 approach in primary human CD34^+^ haematopoietic stem and progenitor cells (HSPCs) showed that using PE7 with a La-accessible pegRNA led to a 5.2-fold improvement of an *HBB* edit compared with PEmax with a La-blocked pegRNA (Fig. 5h). PE7 also enabled 41.0% intended editing efficiency (0.4% indels) at the *ATP1A1* locus compared with 20.5% and 25.5% (0.1% and 0.2% indels, respectively) by PEmax with La-blocked pegRNA and epegRNA, respectively (Extended Data Fig. 11g). These data show proof of principle for leveraging La to optimize prime editing in primary cells.

## Discussion

Through genome-scale genetic screens, we identified La, a small RNA-binding protein, as a strong promoting factor of prime editing. Subsequent characterization showed that endogenous La functionally interacts with the 3′ ends of polyuridylated pegRNAs and promotes the stability and integrity of Pol III-transcribed pegRNAs and epegRNAs. These results complement an emerging understanding that instability of reverse transcription templates limits prime editing efficiency. Previous efforts to mitigate this limitation include adding structured RNA motifs to the 3′ ends of pegRNAs^18,30,34^, as in epegRNAs, and circularizing untethered templates^29,35^. Our results indicated that the role of La might be at least partially redundant with epegRNAs, as epegRNAs buffered *La*-associated phenotypes relative to pegRNAs. However, when editing with PE7, epegRNAs provided no additional benefit over pegRNAs, except in a minority of cases. We therefore expect pairing PE7—which outperformed PEmax in nearly all conditions examined—with pegRNAs to be optimal for many applications.

Our study also highlights how terminal uridines^36–38^ and chemical modification strategies developed to protect synthetic sgRNAs from RNA exonucleases^28^ have been haphazardly added to pegRNAs across studies^5,18,29^. Unlike sgRNAs, which are almost entirely protected by bound Cas9 proteins, pegRNAs rely on exposed 3′ extensions. We therefore cannot expect chemical modification strategies developed for sgRNAs to be optimal or even sufficient for synthetic pegRNAs. Additionally, when combined with commercially recommended chemical modifications for sgRNAs, the addition of 3′ polyU tracts to pegRNAs should allow La binding (3′-mU*mU*mU*U from IDT) or not (3′-mU*mU*mU from Synthego), which may have effects on editing even without using PE7 (for example, see Fig. 5h). For applications that require RNA delivery, we anticipate that pairing PE7 with our La-accessible pegRNAs will be particularly advantageous, especially compared with epegRNAs, which are currently difficult to chemically synthesize owing to their longer length.

Although the exact mechanism (or mechanisms) by which La promotes prime editing and the boundaries within which PE7 provides improvement remain to be fully elucidated (for example, across additional cell types, delivery modalities and editing conditions), our study represents an important first step in understanding this key cellular determinant and exploiting its function for optimization. Many possible avenues also remain for future optimization. For example, design rules for La-accessible pegRNAs could be refined, the linker between PEmax and La(1–194) could be optimized or La(1–194) could be appended to more compact prime editors^39^ to reduce the size of PE7, which is currently only 226 amino acids longer than PEmax (2131 amino acids). Additionally, because ectopic expression of full-length *La* alongside PEmax also improved prime editing (Fig. 2c), systems using in trans overexpression could be explored. Finally, we note that La was first identified as an autoantigen in patients with systemic lupus erythematosus and in patients with Sjogren’s syndrome^2^. Therefore, as with all genome-editing tools, application-specific consequences of PE7 will need to be considered before therapeutic use.

In summary, through the identification and characterization of La as a key cellular determinant of prime editing, our study expanded our understanding of the cellular processes that directly affect prime editing, demonstrated methods for improving prime editing efficiencies and suggested useful avenues for future optimization.

## Methods

### General methods

CRISPRi sgRNAs were cloned into pU6-sgRNA EF1Alpha-puro-T2A-BFP (Addgene, 60955)^13^ as described in https://weissman.wi.mit.edu/resources/sgRNACloningProtocol.pdf (Supplementary Table 4). Plasmids for transfection expressing pegRNAs, epegRNAs and non-CRISPRi sgRNAs were cloned by Gibson Assembly of gene fragments without adapters from Twist Bioscience and pU6-pegRNA-GG-acceptor plasmid (Addgene, 132777)^4^ digested using NdeI or BsaAI/BsaI-HFv2 (New England Biolabs, R0111S, R0531S, R3733S) (Supplementary Table 4). Plasmids for transduction expressing pegRNAs and epegRNAs were cloned by Gibson Assembly of gBlock from Integrated DNA Technologies and pU6-sgRNA EF1Alpha-puro-T2A-BFP digested using BstXI and XhoI (New England Biolabs, R0113S and R0146S) (Supplementary Table 4). The FACS and MCS reporter plasmids were cloned by Gibson Assembly with pALD-lentieGFP-A (Aldevron) as the backbone, IRES2 from pLenti-DsRed_IRES_eGFP (Addgene, 92194)^41^ and the synthetic surface marker from pJT039 (Addgene, 161927)^15^. The AAVS1 PEmax knock-in plasmid was generated by restriction cloning with a backbone plasmid modified from pAAVS1-Nst-MCS (Addgene, 80487)^20^, PEmax editor from pCMV-PEmax (Addgene, 174820)^5^ and IRES2 from pLenti-DsRed_IRES_eGFP. Plasmids of PEmax fused to La or the La N-terminal domain (Supplementary Table 5), including pCMV-PE7 (Addgene, 214812), were generated by restriction cloning using pCMV-PEmax as the backbone (linker A, SGGS×2-XTEN16-SGGS×2; linker B, SGGS×2-bpNLS^SV40^-SGGS×2; linker C, SGGS). pCMV-PE7-P2A-hMLH1dn was cloned by Gibson Assembly with pCMV-PE7 as the backbone and an insert fragment PCR amplified from pCMV-PEmax-P2A-hMLH1dn (Addgene, 174828)^5^. pCMV-PE7-mutant (Q20A, Y23A, Y24F and F35A) was cloned by Gibson Assembly with pCMV-PE7 as the backbone and a mutation-containing gene fragment without adapters from Twist Bioscience. The plasmid for in vitro transcription (IVT) of PE7 mRNA, pT7-PE7 for IVT (Addgene, 214813), was cloned by Gibson Assembly with pT7-PEmax for IVT (Addgene, 178113)^5^ as the backbone and an insert fragment PCR amplified from pCMV-PE7. Lentiviral transfer plasmids expressing PEmax (pWY005/pWY004) or PE7 (pWY008/pWY007) with IRES2-driven eGFP or eGFP-T2A-NeoR as the selectable marker were cloned by Gibson Assembly with pU6-sgRNA EF1Alpha-puro-T2A-BFP as the backbone, UCOE and SFFV promoter from pMH0001 (Addgene, 85969)^42^, IRES2 from pLenti-DsRed_IRES_eGFP and T2A-NeoR from pAAVS1-Nst-MCS. All DNA amplification for molecular cloning was performed using Platinum SuperFi II PCR master mix (Invitrogen, 12368010). All plasmids were extracted using NucleoSpin Plasmid, Mini kits (Macherey-Nagel, 740588.250), ZymoPURE II Plasmid Midiprep kits (Zymo Research, D4201) or EndoFree Plasmid Maxi kits (Qiagen, 12362). Primers were ordered from Integrated DNA Technologies (Supplementary Table 6).

### Flow cytometry and FACS

Flow cytometry data were analysed using BD FACSDiva (8.0.1), Attune Cytometric Software (5.2.0) or FlowCytometryTools (0.5.1; https://github.com/eyurtsev/FlowCytometryTools)^43^. Data from flow cytometry analysis and FACS can be found in Figs. 1c and 2f, Extended Data Figs. 1d–f,h–j, 2a–c,f, 3a,f,g, 4a and 10b,c, Supplementary Figs. 1–7 and Supplementary Table 7.

### In vitro transcription of prime editor mRNA

Prime editor mRNA was in vitro transcribed as previously described^44^. Plasmids with PEmax or PE7 coding sequence flanked by an inactivated T7 promoter, a 5′ untranslated region (UTR) and a Kozak sequence in the upstream as well as a 3′ UTR in the downstream were purchased from Addgene (pT7-PEmax for IVT) or cloned as described above (pT7-PE7 for IVT). In vitro transcription templates were generated by PCR to correct the T7 promoter and to install a 119-nucleotide poly(A) tail downstream of the 3′ UTR. PCR products were purified by DNA Clean & Concentrator-5 (Zymo Research, D4003) and SPRIselect (Beckman Coulter, B23317) for cell line and T cell experiments, respectively, and stored at −20 °C until further use. mRNA was generated using a HiScribe T7 mRNA kit with CleanCap Reagent AG (New England BioLabs, E2080S) for cell line experiments and a HiScribe T7 High Yield RNA Synthesis kit (New England Biolabs, E2040S) in the presence of RNase inhibitor (New England Biolabs, M0314L) and yeast inorganic pyrophosphatase (New England Biolabs, M2403L) for T cell experiments. All mRNA was produced with UTP fully replaced with N^1^-methylpseudouridine-5′-triphosphate (TriLink Biotechnologies, N-1081) and co-transcriptional capping by CleanCap Reagent AG (TriLink Biotechnologies, N-7113). Transcribed mRNA was precipitated by 2.5 M lithium chloride (Invitrogen, AM9480), resuspended in nuclease-free water (Invitrogen, AM9939), quantified by a NanoDrop One UV-Vis spectrophotometer (Thermo Scientific), normalized to 1 μg μl^−1^ and stored at −80 °C. mRNA for T cell experiments was additionally quantified by Agilent 4200 TapeStation. Prime editor mRNA for HSPC experiments was in vitro transcribed as described in the section ‘HSPC isolation, culture and prime editing’.

### General mammalian cell culture conditions

Lenti-X 293T was purchased from Takara (632180). K562 (CCL-243), HeLa (CCL-2) and U2OS (HTB-96) were purchased from the American Type Culture Collection. The K562 CRISPRi cell line constitutively expressing dCas9-BFP-KRAB (pHR-SFFV-dCas9-BFP-KRAB, Addgene, 46911)^12^ was a gift from J. Weissman. Lenti-X 293T, HeLa and U2OS cells were cultured and passaged in Dulbecco’s modified Eagle’s medium (DMEM) (Corning, 10-013-CV), DMEM (Corning, 10-013-CV) and McCoy’s 5A (Modified) medium (Gibco, 16600082) supplemented with 10% (v/v) FBS (Corning, 35-010-CV) and 1× penicillin–streptomycin (Corning, 30-002-CI). For lipofection and nucleofection, 1× penicillin–streptomycin was not supplemented. K562 and K562 CRISPRi cells were cultured and passaged in RPMI 1640 medium (Gibco, 22400089) supplemented with 10% (v/v) FBS (Corning, 35-010-CV) and 1× penicillin–streptomycin–glutamine (Gibco, 10378016). For nucleofection, 1× penicillin–streptomycin–glutamine was replaced by 1× l-glutamine at 292 μg ml^−1^ final concentration (Corning, 25-005-CI). All cell types were incubated, maintained and cultured at 37 °C with 5% CO_2_. Cell lines were authenticated by short tandem repeat profiling and tested negative for mycoplasma.

### Lentivirus packaging and transduction

To package lentiviruses, Lenti-X 293T cells were seeded at 9 × 10^5^ cells per well in 6-well plates (Greiner Bio-One, 657165) and were transfected at 70% confluency. For transfection, 6 μl TransIT-LT1 (Mirus, MIR 2300) was mixed and incubated with 250 μl Opti-MEM I reduced serum medium (Gibco, 31985070) at room temperature for 15 min, then mixed with 100 ng pALD-Rev-A (Aldevron), 100 ng pALD-GagPol-A (Aldevron), 200 ng pALD-VSV-G-A (Aldevron) and 1,500 ng transfer plasmids at room temperature for another 15 min, and was added dropwise to Lenti-X 293T cells followed by gentle swirling for proper mixing. At 10 h after transfection, ViralBoost reagent (ALSTEM, VB100) was added at 1× final concentration. At 48 h after transfection, the virus-containing supernatant was collected, filtered through a 0.45-µm cellulose acetate filter (VWR, 76479-040) and stored at −80 °C. Lentiviruses for CRISPRi screens were similarly packaged with hCRISPRi-v2 library (Addgene, 83969)^14^ as transfer plasmids in 145 mm plates (Greiner Bio-One, 639160). For transduction of K562 cells, cells were resuspended in fresh culture medium supplemented with 8 µg ml^−1^ polybrene (Santa Cruz Biotechnology, sc-134220), mixed with lentivirus-containing supernatant and centrifuged at 1,000*g* at room temperature for 2 h. For transduction of U2OS and HeLa cells, the cell culture was supplemented with 8 µg ml^−1^ polybrene and lentivirus-containing supernatant. The percentages of transduced (positive for the fluorescent protein marker) cells were determined by AttueNXT flow cytometry 72 h after transduction. To generate stably transduced cell lines, cells were selected by 3 μg ml^−1^ puromycin (Goldbio, P-600-100) 48 h after transduction until >95% of live cells were marker positive.

### Construction of FACS reporter cell line and FACS-based genome-scale CRISPRi screen

To construct our FACS reporter cell line, K562 CRISPRi cells were transduced with FACS reporter lentiviruses at a 0.17 multiplicity of infection (m.o.i.; 15.3% infection). The transduced (mCherry^+^) population was isolated using a BD FACSAria Fusion flow cytometer and expanded as the FACS reporter cell line. For the FACS-based genome-scale CRISPRi screen, two replicates were independently performed a day apart. For each replicate, 2.4 × 10^8^ FACS reporter cells were transduced with hCRISPRi-v2 lentiviruses at a 0.29 m.o.i. (25% infection) and were selected by 3 μg ml^−1^ puromycin 48 h after transduction. Seven days after transduction, 3.2 × 10^8^ fully selected cells were nucleofected using the SE Cell Line 4D-Nucleofector X kit L (Lonza, V4XC-1024) and pulse code FF120, according to the manufacturer’s protocol. Each nucleofection consisted of 1 × 10^7^ cells, 7,500 ng pCMV-SaPE2 (Addgene, 174817)^5^, 2,500 ng +7 GG-to-CA pegRNA plasmid and 833 ng +50 nicking sgRNA plasmid. Three days after nucleofection, 1.5 × 10^8^ cells were sorted using a BD FACSAria Fusion flow cytometer. Specifically, cells were first gated on mCherry^+^ and BFP^+^, of which eGFP^+^ and eGFP^–^ populations were collected. gDNA was extracted from both populations using a NucleoSpin Blood XL Maxi kit (Macherey-Nagel, 740950.50). The entirety of gDNA from both populations was used for PCR amplification of integrated hCRISPRi-v2 sgRNAs. Each 100 μl PCR reaction was performed with 10 μg of gDNA, 1 μM of forward primer that anneals in the mouse U6 promoter, 1 μM of reverse primer that anneals to the sgRNA constant region, and 50 μl of NEBNext Ultra II Q5 master mix (New England BioLabs, M0544X) with the following cycling conditions: 98 °C for 30 s, 23 cycles of (98 °C for 10 s, 65 °C for 75 s), followed by 65 °C for 5 min. The PCR product was purified using SPRIselect (Beckman Coulter, B23318) with a double size selection (0.65× right side and 1.35× left side), quantified using a Qubit 1× dsDNA High Sensitivity kit (Invitrogen, Q33231) and a high-sensitivity DNA chip (Agilent Technologies, 5067-4626) on an Agilent 2100 Bioanalyzer, and sequenced using a NovaSeq 6000 SP Reagent kit (v.1.5) for 100 cycles (Illumina, 20028401) with 50 cycles for the R1 read with a custom sequencing primer and 8 cycles for the i7 index read.

### Construction of the MCS reporter cell line and MCS-based genome-scale CRISPRi screen

To construct our MCS reporter cell line, K562 CRISPRi cells were transduced with MCS reporter lentiviruses at a 0.09 m.o.i. (8.5% infection). The transduced (eGFP^+^) population was isolated using a BD FACSAria Fusion flow cytometer and expanded as the MCS reporter cell line. MCS-based genome-scale CRISPRi screens with +7 GG-to-CA PE3+50, PE4 and PE5+50 edits were performed in parallel with two replicates each. A total of 2.1 × 10^8^ MCS reporter cells were transduced with hCRISPRi-v2 lentiviruses at a 0.16 m.o.i. (15% infection) for all screen conditions and were selected by 3 μg ml^−1^ puromycin 48 h after transduction. Seven days after transduction, 1 × 10^8^ fully selected cells were nucleofected for each replicate of each edit using the SE Cell Line 4D-Nucleofector X kit L (Lonza, V4XC-1024) and pulse code FF120, according to the manufacturer’s protocol. Each nucleofection consisted of 1 × 10^7^ cells and varying amounts of plasmids encoding prime editing components. Specifically, for PE2 and PE3, 7,500 ng pCMV-SaPE2, 2,500 ng +7 GG-to-CA pegRNA plasmid, 833 ng +50 nicking sgRNA plasmid (PE3) were used per nucleofection. For PE4 and PE5, 6,000 ng pCMV-SaPE2, 3,000 ng pEF1a-hMLH1dn (Addgene, 174823)^5^, 2,000 ng +7 GG-to-CA pegRNA plasmid and 667 ng +50 nicking sgRNA plasmid (PE5) were used. Four days after nucleofection, cells from each replicate and condition were magnetically separated into bead-bound and unbound fractions as previously described^15^. The gDNA extraction, PCR, NGS library quality control and sequencing were performed as described in the section above. We note that the MCS reporter was less efficient in cell separation than the FACS reporter (Extended Data Fig. 1f,g), which is possibly due to the failure to remove dead cells, debris or doublets from the bead-bound or unbound fraction.

### Analysis of genome-scale CRISPRi screen

Sequencing reads were aligned to the hCRISPRi-v2 library (five sgRNAs per gene) using custom Python (2.7.18) scripts as previously described^14^ (scripts available at GitHub (https://github.com/mhorlbeck/ScreenProcessing)^45^). sgRNA-level phenotypes were calculated as the log_2_ enrichment of normalized read counts (sgRNA counts normalized to the total count from the sample and relative to the median of non-targeting controls) within populations of marker-positive cells (GFP^+^ or bead-bound) compared with marker-negative cells (GFP^–^ or bead-unbound) (Supplementary Table 1). Before calculation, a read count minimum of 50 was imposed for each sgRNA within each sample. Gene-level phenotypes were then calculated for each annotated transcription start site by averaging the phenotypes of the strongest 3 sgRNAs by absolute value. Negative control pseudogenes were generated by random sampling, assigning five non-targeting sgRNAs to each pseudogene. sgRNA-level phenotypes were used as input to the CRISPhieRmix (v.0.1.0)^16^ under default parameters with *µ* = 2 to formally evaluate the effect each gene has on prime editing efficiency (Supplementary Tables 2 and 3). Screen results were plotted using R (4.2.2) and ggplot2 (3.4.1).

### Considerations regarding the design of our prime editing reporter system

The reporter assays used for our genome-scale CRISPRi screens were designed with two primary considerations: scale and phenotype.

#### Scale

We developed our reporter system to perform cost-effective, high-throughput prime editing screens. Although easy to implement and scale, reporter screens are always limited in their ability to identify genes with subtle phenotypes owing to their reliance on low-resolution readouts—especially compared with screens performed with molecular readouts (for example, Repair-seq^5^). Our prime editing reporter assays should therefore be considered a scalable means of identifying strong prime editing regulators. Additionally, owing to lower technical variability observed in data from the FACS-based screen, hits from that screen should be considered higher priority candidates than those from our MCS-based screens.

Our FACS-based screen identified 36 hit genes (35 negative regulators and 1 positive regulator, FDR ≤ 0.01). Although this rate of hit identification is lower than typically observed in genome-scale screens designed to interrogate cellular processes, prime editing is a synthetic system, and cellular regulators, although present and important, are therefore not expected to be abundant. Indeed, previously performed Repair-seq screens identified only 10 sgRNAs against 4 genes with >2-fold change in similarly implemented PE3-based editing (out of 476 DNA repair associated genes)^5^. The paucity of hits over this >2-fold threshold was therefore expected in our screens, but combined with the fact that our screens were designed to identify only strong regulators, correlations between screen replicates were expectedly low. Pearson correlation coefficients for replicate sgRNA-level phenotypes were 0.053 (FACS, PE3), 0.042 (MCS, PE3), 0.058 (MCS, PE4) and 0.054 (MCS, PE5). For replicate gene-level phenotypes, correlation coefficients were 0.125 (FACS, PE3), 0.071 (MCS, PE3), 0.090 (MCS, PE4) and 0.073 (MCS, PE5).

#### Phenotype

When validating our prime editing reporter constructs, we observed enrichment of outcomes containing only intended edits and enrichment of outcomes with intended edits and accompanying indels among marker-positive cells (that is, GFP^+^ FACS reporter cells isolated by flow cytometry or MCS reporter cells bound to protein G beads) (Extended Data Fig. 1f,g,i). Accumulation of both types of outcomes within our marker-positive populations reflected a design choice. Specifically, we designed the target site in our reporters such that PE3-induced indels, which typically fall between the primary and complementary strand nicks^5^, would not frequently disrupt the open reading frame of the reporter genes and therefore would not prevent marker expression induced by a concomitantly installed intended edit (Fig. 1b). Phenotypes from this reporter system therefore represent overall frequencies of editing outcomes with the intended edit, but not the homogeneity of editing outcomes within marker-positive populations.

### Tissue culture transfection and transduction protocols and gDNA extraction

For *La* knockdown in Lenti-X 293T by siRNA reverse transfection, 120 pmole ON-TARGETplus Human *SSB* siRNA (Horizon, LQ-006877-01-0005) or ON-TARGETplus Non-targeting Control Pool (Horizon, D-001810-10-05) were mixed thoroughly with 500 μl Opti-MEM I reduced serum medium (Gibco, 31985070) and 4 μl Lipofectamine RNAiMAX transfection reagent (Invitrogen, 13778150) in each well of 6-well plates (Greiner Bio-One, 657165), incubated at room temperature for 15 min before 4 × 10^5^ Lenti-X 293T cells in 2.5 ml penicillin–streptomycin-free medium were added. The reverse transfected cells were used for RT–qPCR or downstream prime editing experiments as described in the corresponding Methods sections.

For prime editing in Lenti-X 293T cells by plasmid transfection, 18,000 cells were seeded in 100 μl penicillin–streptomycin-free medium per well in 96-well plates (Nunc, 167008). At 18 h after seeding, a 10 μl mixture of 200 ng pCMV-PE2 (Addgene, 132775)^4^, 66 ng pegRNA, 22 ng nicking sgRNA, 0.5 μl Lipofectamine 2000 transfection reagent (Invitrogen, 11668027) and Opti-MEM I reduced serum medium (Gibco, 31985070) was incubated at room temperature for 15 min and added to each well. At 72 h after transfection, the culture medium was removed, cells were washed with DPBS (Gibco, 14190144) and gDNA was extracted by adding 40 μl freshly prepared lysis buffer into each well. The lysis buffer consisted of 10 mM Tris pH 8.0 (Gibco, AM9855G), 0.05% SDS (Invitrogen, 15553027), 25 μg ml^−1^ proteinase K (Invitrogen, AM2546) and nuclease-free water (Invitrogen, AM9939). The gDNA extract was incubated at 37 °C for 90 min and then transferred into PCR strips (USA Scientific, 1402-4700) for 80 °C inactivation of proteinase K for 30 min in a Bio-Rad T100 thermal cycler.

For prime editing in Lenti-X 293T, HeLa and U2OS cells by plasmid nucleofection, 750 ng prime editor plasmid, 250 ng pegRNA plasmid and 83 ng nicking sgRNA plasmid (PE3 and PE5) were nucleofected. For each sample, 2 × 10^5^ LentiX-293T cells, 1 × 10^5^ HeLa cells or 1 × 10^5^ U2OS cells were nucleofected using SF (Lonza, V4XC-2032), SE (Lonza, V4XC-1032) and SE Cell Line 4D-Nucleofector X kit S with program CM-130, CN-114 and DN-100, respectively, according to the manufacturer’s protocols. PE4 and PE5 experiments in U2OS cells were performed with pCMV-PEmax-P2A-hMLH1dn and pCMV-PE7-P2A-hMLH1dn editor plasmids. After nucleofection, cells were cultured in 24-well plates (Greiner Bio-One, 662165), and the culture medium was removed 72 h after nucleofection. Cells were washed with DPBS (Gibco, 14190144) and gDNA was extracted by adding 110 μl freshly prepared lysis buffer (described above) into each well. The gDNA extract was incubated at 37 °C for 90 min and transferred into PCR strips (USA Scientific, 1402-4700) for 80 °C inactivation of proteinase K for 40 min in a Bio-Rad T100 thermal cycler.

For nucleofections in K562 cells (except those for CRISPRi screens, AAVS1 knock-in, *La* knockout, small RNA sequencing and RNA sequencing), 1 × 10^6^ cells were nucleofected with specified amounts of plasmids or synthetic guide RNAs using the SE Cell Line 4D-Nucleofector X kit S (Lonza, V4XC-1032) and program FF-120, according to the manufacturer’s protocol. For testing FACS-reporter and MCS-reporter and validation of *La* phenotype in reporter cell lines, 900 ng pCMV-SaPE2, 300 ng pegRNA plasmid, 100 ng nicking sgRNA plasmid (PE3 and PE5) and 450 ng pEF1a-hMLH1dn (PE4 and PE5) were nucleofected. For validation of *La* phenotype in K562 PEmax parental and *La* knockout clones, 500 ng pegRNA plasmid was nucleofected. For rescue experiments, 500 ng pegRNA plasmid and 1,000 ng plasmid encoding La, La mutants or mRFP control were nucleofected. For SaCas9 cutting in MCS reporter cells, 800 ng pX600 (Addgene, 61592)^21^ and 400 ng +7 GG-to-CA pegRNA plasmid were nucleofected. For SaPE2 editing using the PE4 approach in K562 PEmax parental and La-ko4 cells, 800 ng pCMV-SaPE2, 400 ng pegRNA plasmid and 400 ng pEF1a-hMLH1dn were nucleofected. For SaCas9, SaBE4 and SaABE8e editing in K562 PEmax parental and La-ko4 cells, 400 ng pegRNA or sgRNA plasmid and 800 ng pX600, SaBE4-Gam (Addgene, 100809)^23^ or SaABE8e (Addgene, 138500)^24^ were nucleofected. Synthetic pegRNAs and a nicking sgRNA with specified sequences and chemical modifications were ordered as Custom Alt-R gRNA from Integrated DNA Technologies (Supplementary Table 8). According to an incremental titration of a *DNMT1* +5 G-to-T no-polyU synthetic pegRNA in K562 PEmax parental cells, intended editing efficiencies were already saturated at 100 pmole input (Extended Data Fig. 5b). Therefore, 100 pmole synthetic pegRNA and 50 pmole nicking sgRNA (PE3) were used for nucleofection unless otherwise specified. At 72 h after nucleofection, 1 × 10^6^–2 × 10^6^ cells were collected in 1.5 ml tubes (Eppendorf, 0030123611), washed with 1 ml DPBS (Gibco, 14190144) and resuspended in 100 μl freshly prepared lysis buffer described above. The gDNA extract was incubated at 37 °C for 120 min and transferred into PCR strips (USA Scientific, 1402-4700) for 80 °C inactivation of proteinase K for 40 min in a Bio-Rad T100 thermal cycler.

For prime editing in K562 and U2OS cells using editor mRNA and synthetic pegRNA, 1 × 10^6^ K562 and 1 × 10^5^ U2OS cells were nucleofected with 1 µg editor mRNA and 50 pmole synthetic pegRNA using the SE Cell Line 4D-Nucleofector X kit S (Lonza, V4XC-1032) with program FF-120 and DN-100, respectively, according to the manufacturer’s protocols. After nucleofection, cells were cultured for 72 h and collected for gDNA extract.

For prime editing in HeLa and U2OS cells by lentiviral delivery of pegRNAs or epegRNAs and nucleofection of editor plasmids or mRNA, cells were transduced with lentiviruses expressing pegRNAs or epegRNAs (20–40% infection) and were fully selected by 3 μg ml^−1^ puromycin. 1 × 10^5^ stably transduced HeLa and U2OS cells were nucleofected with 750 ng editor plasmid or 1 µg editor mRNA using the SE Cell Line 4D-Nucleofector X kit S (Lonza, V4XC-1032) with program CN-114 and DN-100, respectively, according to the manufacturer’s protocols. After nucleofection, cells were cultured for 72 h and collected for gDNA extract.

For prime editing in K562 cells by lentiviral delivery of prime editors and pegRNAs or epegRNAs, K562 cells were transduced with lentiviruses expressing PEmax or PE7 (with IRES2-driven eGFP or eGFP-T2A-NeoR as the selectable marker). The transduced populations (eGFP^+^, 20–30%) were isolated using a BD FACSAria Fusion flow cytometer 9 days after transduction, further transduced with lentiviruses expressing pegRNAs or epegRNAs (approximately 50% infection), fully selected by 3 μg ml^−1^ puromycin and collected 11 days after the second transduction for gDNA extract.

### Amplicon sequencing

gDNA sequences containing target sites were amplified through two rounds of PCR reactions (PCR1 and PCR2). In PCR1, genomic regions of interest were amplified with primers containing forward and reverse adapters for Illumina sequencing. Each 20 μl PCR1 reaction consisted of 1–2 μl gDNA extract, 0.5 µM of each forward and reverse primer, 10 μl Phusion U Green Multiplex PCR master mix (Thermo Scientific, F564L) and nuclease-free water (Invitrogen, AM9939) and was performed with the following cycling conditions: 98 °C for 2 min, 28 cycles of (98 °C for 10 s, 61 °C for 20 s, and 72 °C for 30 s), followed by 72 °C for 2 min. Successful PCR1 amplification was confirmed by 1% agarose (Goldbio, A-201-100) gel electrophoresis before proceeding to PCR2 to uniquely index each sample. Each 14 µl PCR2 reaction consisted of 1 µl unpurified PCR1 product, 0.5 µM of each forward and reverse Illumina barcoding primer, 7 μl Phusion U Green Multiplex PCR master mix (Thermo Scientific, F564L) and nuclease-free water (Invitrogen, AM9939) and was performed with the following cycling conditions: 98 °C for 2 min, 9 cycles of (98 °C for 10 s, 61 °C for 20 s, and 72 °C for 30 s), followed by 72 °C for 2 min. Successful PCR2 amplification was confirmed by 1% agarose gel electrophoresis before reactions were pooled by common amplicons. A total of 30 µl pooled PCR2 reactions of each common amplicon was purified by 1% agarose gel electrophoresis with a manual size selection of 200–600 bp according to a 100 bp DNA ladder (Goldbio, D001-500), extracted using the Zymoclean Gel DNA Recovery kit (Zymo Research, D4001) and eluted in 30 µl buffer EB (Qiagen, 19086). The gel-purified PCR2 products were quantified using a Qubit 1× dsDNA High Sensitivity kit (Invitrogen, Q33231) and a high-sensitivity DNA chip (Agilent Technologies, 5067-4626) on an Agilent 2100 Bioanalyzer and sequenced using the MiSeq Reagent Micro kit v2 300 cycles (Illumina, MS-103-1002) or Nano kit v2 300 cycles (Illumina, MS-103-1001) with 300 cycles for the R1 read, 8 cycles for the i7 index read and 8 cycles for the i5 index read. Sequencing reads were demultiplexed through HTSEQ (Princeton University High Throughput Sequencing Database, https://htseq.princeton.edu/) and sequencing adapters were trimmed using Cutadapt (4.1)^46^.

To quantify prime editing outcomes, amplicon sequencing reads were aligned to the corresponding reference sequence (Supplementary Table 9) with CRISPResso2 (2.2.11)^47^ in HDR batch mode using the intended editing outcome as the expected allele (“-e”) with the parameters “-q 30”, “--discard_indel_reads”, and with the quantification window centred at the pegRNA nick (“-wc −3”). The quantification window sizes (“-w”) are specified in Supplementary Table 7^4,5,18^. The frequency of intended editing without indels was calculated as follows: (number of non-discarded HDR-aligned reads)/(number of reads that aligned all amplicons). The frequency of intended editing with indels was calculated as follows: (number of discarded HDR-aligned reads)/(number of reads that aligned all amplicons). The frequency of total intended editing (with or without indels) was calculated as (number of HDR-aligned reads)/(number of reads that aligned all amplicons). The frequency of total indels was calculated as follows: (number of discarded reads)/(number of reads that aligned all amplicons). The frequency of indels without intended editing was calculated as (number of discarded reference-aligned reads)/(number of reads that aligned all amplicons). Throughout, we refer to ‘intended edit’ efficiencies as the frequencies of intended editing without indels and ‘indel’ efficiencies as the frequencies of total indels (with and without the intended edit) in this study unless otherwise specified. In Figs. 2b,c, 3b,d, 4c,f and 5a,c,d,f,h and Extended Data Figs. 3b,h, 5c–e, 9a,b, 10a and 11a,d,f,g, the indel frequency is included for each sample adjacent to the corresponding intended editing efficiency.

To quantify off-target prime editing, two to four of the most common Cas9 off-target sites experimentally determined^32^ for each on-target locus were amplified from gDNA extracts of U2OS cells nucleofected with plasmids encoding PEmax or PE7 and pegRNAs targeting HEK3, HEK4, *FANCF* and *EMX1* loci in Fig. 4c. Off-target editing was quantified as previously described with minor modifications^4,5,18^. Specifically, reads were aligned to corresponding off-target reference sequences using CRISPResso2 (2.2.11) in standard batch mode with parameters “-q 30”, “-w 10” and “--discard_indel_reads”. Each off-target amplicon sequence was compared with the 3′ DNA flap sequence encoded by the pegRNA extension starting from the nucleotide 3′ of Cas9 nick to the downstream until reaching the first nucleotide on the off-target amplicon that is different from the 3′ DNA flap. Any reads with this nucleotide converted to that on the 3′ DNA flap were considered off-target reads and the number of such reads can be found in the output file ‘Nucleotide_frequency_summary_around_sgRNA’. Off-target editing efficiencies were calculated as (number of off-target reads + number of indel-containing reads)/(number of reads that aligned all amplicons).

To quantify Cas9 cutting outcomes, CRISPResso2 (2.2.11) was run in standard batch mode with the parameters “-q 30” and “--discard_indel_reads”. The intended editing efficiency referred to the frequency of indels that was calculated as follows: (number of discarded reference-aligned reads)/(number of reads that aligned all amplicons). Base editing outcomes were quantified using CRISPResso2 (2.2.11) as previously described^23,24^.

### RT–qPCR

To quantify knockdown efficiencies of *La*-targeting CRISPRi sgRNAs in MCS reporter cells or *La* siRNA in Lenti-X 293T cells, total RNA was extracted using a Quick-RNA Miniprep kit (Zymo Research, R1054) with DNase I treatment and 1 µg total RNA was converted to cDNA with SuperScript IV First-Strand Synthesis system (Invitrogen, 18091050) according to the manufacturer’s protocol. Each 20 µl RT–qPCR reaction consisted of 2 µl cDNA, 0.3 µM of each forward and reverse primer, 10 μl SYBR Green PCR master mix (Applied Biosystems, 4309155) and nuclease-free water (Invitrogen, AM9939) and was performed in triplicate on a ViiA 7 Real-Time PCR system (Applied Biosystems) with the following cycling conditions: 50 °C for 2 min, 95 °C for 10 min, and 40 cycles of (95 °C for 15 s, 60 °C for 1 min). Relative *La* expression levels were calculated using the ${2}^{-\Delta \Delta {C}_{{\rm{T}}}}$ method^48^ with *ACTB* (a housekeeping gene) as the internal control in comparison to a non-targeting sgRNA or a non-targeting control siRNA pool.

### Generation of K562 clones with PEmax knock-in at AAVS1

A total of 91.5 pmole Alt-R S.p. Cas9 Nuclease V3 (Integrated DNA Technologies, 1081058) and 150 pmole custom Alt-R gRNA targeting AAVS1^20^ (Integrated DNA Technologies) (Supplementary Table 8) were complexed for 20 min at room temperature and were nucleofected together with 2,000 ng AAVS1 PEmax knock-in plasmid as the HDR template into 7.5 × 10^5^ K562 cells using the SE Cell Line 4D-Nucleofector X kit (Lonza, V4XC-1032) and program FF-120, according to the manufacturer’s protocol. Four days after nucleofection, cells were selected using 400 μg ml^−1^ geneticin (Gibco, 10131027) for 2 weeks before sorted using a BD FACSAria Fusion flow cytometer into 96-well plates at 1 cell per well with 150 μl conditioned culture medium. Single cells were grown and expanded for 2–3 weeks into clonal lines, from which the one with the highest and most homogenous eGFP expression by AttueNXT flow cytometry analysis was selected as the K562 PEmax parental cell line.

### Generation of *La* knockout K562 PEmax cells

A total of 122 pmole Alt-R S.p. Cas9 Nuclease V3 (Integrated DNA Technologies, 1081058) and 200 pmole Alt-R CRISPR-Cas9 sgRNA targeting *La* (Integrated DNA Technologies, Hs.Cas9.SSB.1.AA) (Supplementary Table 8) were complexed for 20 min at room temperature and were nucleofected into 5 × 10^5^ K562 PEmax parental cells using the SE Cell Line 4D-Nucleofector X kit (Lonza, V4XC-1032) and program FF-120, according to the manufacturer’s protocol. Five days after nucleofection, cells were sorted using a BD FACSAria Fusion flow cytometer into 96-well plates at 1 cell per well with 150 μl conditioned culture medium. Single cells were grown and expanded for 2–3 weeks into clonal lines. Clones with high eGFP^+^ cell% according to AttueNXT flow cytometry analysis were selected for further characterization by targeted sequencing at the genomic *La* (*SSB*) locus and CRISPResso2 (2.2.11) analysis. For each experiment involving K562 PEmax parental cells and derived *La* knockout cells, eGFP^+^ cell percentage of each cell line was quantified by flow cytometry before transfection (Supplementary Table 7).

### Western blotting

Cells were washed with DPBS (Gibco, 14190144), lysed in 2× western lysis buffer, boiled for 5 min at 95 °C and stored at −80 °C before use. For SDS–PAGE, samples were reheated at 95 °C for 5 min, thoroughly mixed, loaded to a 10% gel and run for 1.5 h at 150 V. Precision Plus Protein Dual Color standards (Bio-Rad, 161-0374) was loaded as the marker. The proteins were transferred into a nitrocellulose membrane (VWR, 10120-060) using a Trans-Blot SD semi-dry transfer cell (Bio-Rad). Antibodies were diluted in 5% Blotto (5% nonfat dry milk in TBST) and incubated with the membrane for 1 h at room temperature. The same membrane was sequentially immunoblotted with the following primary antibodies: anti-La mouse monoclonal antibody (1:5,000; Abcam, ab75927), anti-GAPDH rabbit monoclonal antibody (1:5,000; Abcam, ab181602) and Guide-it Cas9 rabbit polyclonal antibody (1:1,000; Takara, 632607). The following secondary antibodies were used: HRP-conjugated sheep anti-mouse polyclonal antibody (1:2,000; VWR, 95017-332) and HRP-conjugated donkey anti-rabbit polyclonal antibody (1:2,000; VWR, 95017-556). After incubating with secondary antibodies, the membrane was washed with TBST and immersed into Lumi-LightPLUS western blotting substrate (Sigma, 12015196001) for 3 min in the dark before exposure. The blotting results were developed with films (SpCas9 not imaged with this technique) and/or taken with Azure Biosystems 600. The Restore Western Blot Stripping buffer (Thermo Scientific, 21059) was applied to strip the membrane before reprobing. Cropped portions of western blot analyses are presented in Fig. 2a and Extended Data Fig. 3d. Uncropped images and imaging details are provided in Supplementary Fig. 8.

### Cell growth assay

To quantify the effect of *La* knockout on cell growth, K562 PEmax parental, La-ko4, and La-ko5 cells were monitored using AttueNXT flow cytometry with three individual replicates per cell line and each replicate in a 100 mm cell culture dish (Greiner Bio-One, 664160). On each day, live cell density (average of three repeat measurements) of each replicate and each cell line was quantified by flow cytometry, diluted to approximately 5 × 10^5^ cells per ml and quantified again immediately and 24 h after dilution. The cell doubling was calculated as the ratio of live cell density measured 24 h after dilution to that measured immediately after dilution in log_2_ scale.

### Small RNA sequencing

Small RNA sequencing with targeting pegRNAs and epegRNAs was performed in triplicate and for each replicate, 5 × 10^6^ K562 PEmax parental or La-ko4 cells were nucleofected with 2,500 ng either one of the two pegRNA and epegRNA plasmid sets (set 1 and set 2) using the SE Cell Line 4D-Nucleofector X kit L (Lonza, V4XC-1024) and pulse code FF120, according to the manufacturer’s protocol. Set 1 consisted of plasmids encoding *FANCF* +5 G-to-T pegRNA, HEK3 +1 T-to-A pegRNA, *DNMT1* +5 G-to-T pegRNA, *RUNX1* +5 G-to-T epegRNA (evopreQ_1_), *VEGFA* +5 G-to-T pegRNA and *EMX1* +5 G-to-T epegRNA (mpknot). Set 2 consisted of plasmids encoding *RNF2* +1 C-to-A pegRNA, HEK3 +1 T-to-A epegRNA (mpknot), *DNMT1* +5 G-to-T epegRNA (evopreQ_1_), *RUNX1* +5 G-to-T pegRNA, *VEGFA* +5 G-to-T pegRNA and *EMX1* +5 G-to-T pegRNA. The *VEGFA* +5 G-to-T pegRNA plasmid was shared by both sets and served as the internal control for potential cross-set normalization. The *FANCF* +5 G-to-T pegRNA plasmid and the *RNF2* +1 C-to-A pegRNA were specific to set 1 and 2, respectively. For HEK3, *DNMT1*, *RUNX1* and *EMX1* genomic loci, one set had the pegRNA plasmid whereas the other set had the epegRNA plasmid encoding the same prime edit. Each set only had one evopreQ_1_ epegRNA plasmid and one mpknot epegRNA plasmid. The sets were formulated so that each pegRNA or epegRNA transcript from cells nucleofected with one set could be aligned uniquely to the corresponding pegRNA or epegRNA in that set, based on the observation in preliminary experiments that few fragments were solely mapped to the sgRNA scaffold shared by different pegRNAs and epegRNAs.

Small RNA sequencing with non-targeting *mus DNMT1* (*mDNMT1*) +6 G-to-C pegRNA or epegRNA (tevopreQ_1_) was performed in quadruplicate, and for each replicate, 5 × 10^6^ K562 PEmax parental or La-ko4 cells were nucleofected with 5,000 ng pegRNA or epegRNA plasmid using the SE Cell Line 4D-Nucleofector X kit L (Lonza, V4XC-1024) and pulse code FF120, according to the manufacturer’s protocol.

In both experiments, half of the cells from each nucleofection were collected 24 and 48 h after nucleofection, and total RNA was extracted using the mirVana miRNA Isolation kit with phenol (Invitrogen, AM1560) and was quantified using a NanoDrop One UV-Vis spectrophotometer (Thermo Scientific). For each sample, a small RNA library was constructed with 1 μg total RNA as the input using NEBNext Multiplex Small RNA Library Prep Set for Illumina (set 1) (New England Biolabs, E7300S) and NEBNext Multiplex Oligos for Illumina Index Primers Set 3 (New England Biolabs, E7710S) and Set 4 (New England Biolabs, E7730S) according to the manufacturer’s protocol. Equivolume libraries of all samples were pooled, purified using SPRIselect (Beckman Coulter, B23318) with a double size selection (0.5× right side and 1.35× left side), quantified using a Qubit 1× dsDNA High Sensitivity kit (Invitrogen, Q33231) and a high-sensitivity DNA chip (Agilent Technologies, 5067-4626) on an Agilent 2100 Bioanalyzer, and sequenced using a NovaSeq 6000 SP Reagent kit v.1.5 100 cycles (Illumina, 20028401) with 40 cycles for the R1 read, 8 cycles for the i7 index read and 90 cycles for the R2 read.

To validate *La* phenotype with non-targeting *mDNMT1* +6 G-to-C pegRNA or epegRNA, K562 PEmax parental and La-ko4 cells were transduced with lentiviruses harbouring a target site adapted from *mDNMT1*. Overall, 1 × 10^6^ each transduced cells were nucleofected with 500 or 1,000 ng pegRNA or epegRNA plasmid using the SE Cell Line 4D-Nucleofector X kit S (Lonza, V4XC-1032) and program FF-120, according to the manufacturer’s protocol. One quarter of the number of cells from each nucleofection were collected 1, 2, 3 and 4 days after nucleofection, and the editing outcomes were quantified by amplicon sequencing and CRISPResso2 (2.2.11) analysis.

### Small RNA sequencing data analysis

Sequencing reads were demultiplexed through HTSEQ (Princeton University High Throughput Sequencing Database (https://htseq.princeton.edu/)). The reads were trimmed, aligned and processed using a Snakemake (7.32.4) workflow^49^ and R (4.3.2) (scripts available at Zenodo (10.5281/zenodo.10553303)^50^ or at GitHub (https://github.com/Princeton-LSI-ResearchComputing/PE-small-RNA-seq-analysis)^51^).

Adapters were trimmed using Cutadapt (4.1) -a AGATCGGAAGAGCACACGTCTGAACTCCAGTCAC -A GATCGTCGGACTGTAGAACTCTGAACGTGTAGATCTCGGTGGTCGCCGTATCATT. The trimmed reads were then aligned to the appropriate reference sequences (pegRNAs or epegRNAs) using Bowtie2 (2.5.0)^52^ with default alignment options. Reads that did not align to the appropriate reference (or references) were then aligned to the human genome (GRCh38 primary assembly from Ensembl release 107^53^) using Bowtie2 (2.5.0) with default alignment parameters. Downstream analysis of the alignments used only reads mapped in proper pair, ensuring both ends of the sequenced fragment were properly mapped. Each of such read defines an RNA fragment originating from an RNA molecule for which the sequence was determined by the alignment.

Quantifications of human small RNA, including assigning fragments to human transcripts, genes and biotypes (GENCODE gene annotation release 43)^54^, as well as counting, were performed on properly paired alignments using a custom Python (3.11) script available in the Zenodo or GitHub repository (links provided above). To distinguish between overlapping annotations, each aligned fragment was assigned to the annotation that most closely matched the start and end point of the fragment. The pegRNAs and epegRNAs were quantified for each sample by assigning each properly aligned fragment into one of three bins defined in Supplementary Discussion (*cis*-active, *trans*-active and inactive) using Rsamtools (2.16.0)^55^ and plyranges (1.20.0)^56^. Differential expression was calculated using DESeq2 (1.38.3)^33^ with a design consisting of two covariates: pegRNA and epegRNA plasmid set nucleofected (set 1 or 2) and cell line (K562 PEmax parental or La-ko4). Default parameters were used to estimate library size factors, gene-wise dispersion and fitting of the negative binomial GLM to determine log_2_ fold change values. The log fold change shrinkage was performed using the apeglm algorithm (1.22.1)^57^. The default two-sided Wald test was used to determine the *P* values and the Bonferroni Holm method was used for multiple test correction. Coverage plots were generated using ggplot2 (3.4.4) on data organized using the readr (2.1.4), dplyr (1.1.3), tidyr (1.3.0) and stringr (1.5.0) packages^58^.

For initial quality control of the small RNA sequencing data with targeting pegRNAs and epegRNAs, the following three metrics were calculated: (1) the minimum percentage of pegRNA or epegRNA mapping paired-end reads properly aligned and defined as ‘fragments’ for any sample (98.9%); (2) the minimum percentage of pegRNA or epegRNA fragments uniquely mapped to any one of the 11 pegRNAs and epegRNAs for any sample (94.7%); (3) the minimum percentage of uniquely mapped pegRNA or epegRNA fragments that map to the sense strand of pegRNA or epegRNA for any sample (96.9%). The last metric confirms sequencing of RNA rather than any potential DNA contaminant.

### RNA sequencing and data analysis

Each condition of RNA sequencing was performed in quadruplicate, and for each replicate, 1 × 10^6^ K562 cells were nucleofected with 750 ng PEmax, PE7 or PE7 mutant plasmid and 250 ng pegRNA plasmid encoding HEK3 +1 T-to-A or *PRNP* +6 G-to-T using the SE Cell Line 4D-Nucleofector X kit S (Lonza, V4XC-1032) with program FF-120, according to the manufacturer’s protocols. Nucleofected cells were cultured in 6-well plates with 2.5 ml medium per well. At 24, 48 and 72 h after nucleofection, 150 µl cell culture from each replicate and condition was analysed by AttueNXT flow cytometry to quantify cell viability and live cell density. At 72 h after nucleofection, 1 ml cell culture from each replicate and condition was collected for gDNA extract to quantify prime editing outcomes at the HEK3 or *PRNP* locus. The remaining 1 ml cell culture was pelleted and washed with DPBS (Gibco, 14190144) for total RNA extraction using a RNeasy Plus Mini kit (Qiagen, 74134) with on column DNase I treatment. Total RNA was quantified using a NanoDrop One UV-Vis spectrophotometer (Thermo Scientific) and RNA 6000 Pico chips (Agilent Technologies, 5067-1513) on an Agilent 2100 Bioanalyzer. 3′ mRNA SMART-seq libraries were prepared using total RNA as input on an Apollo NGS library prep system (Takara) following the manufacturer’s protocol. Sequencing libraries were pooled, quantified using a Qubit 1× dsDNA High Sensitivity kit (Invitrogen, Q33231) and a high-sensitivity DNA chip (Agilent Technologies, 5067-4626) on an Agilent 2100 Bioanalyzer and sequenced using a NovaSeq 6000 SP Reagent kit v.1.5 100 cycles (Illumina, 20028401) with 112 cycles for the R1 read and 10 cycles for the index read.

Sequencing reads were demultiplexed through HTSEQ (Princeton University High Throughput Sequencing Database (https://htseq.princeton.edu/)). Alignment, quantification and differential expression were performed using a Snakemake (7.32.3) workflow and R (4.3.1) (scripts available at Zenodo (10.5281/zenodo.10553340)^59^ or GitHub (https://github.com/Princeton-LSI-ResearchComputing/PE-mRNA-seq-diffexp)^60^). The reads were aligned to the GRCh38 genome from Ensembl release 100^53^ using STAR (2.7)^61^ with default alignment parameters. Quantification was performed by STAR during alignment. Differential expression between editors was performed separately for each pegRNA. The standard DESeq2 (1.38) procedure was performed to determine the differential expression between each editor within the set of samples for each pegRNA. Fold changes for lowly expressed genes were shrunken using the adaptive shrinkage estimator from the ashr package (2.2_54)^62^. Figures were generated using R (4.3.1) packages ggplot2 (3.4.3) and ggpubr (0.6.0)^58^. Differential expression analysis results are available in Supplementary Table 10.

### T cell isolation, culture and prime editing

Human peripheral blood Leukopaks enriched for peripheral blood mononuclear cells were sourced from StemCell (StemCell Technologies, 200-0092) with approved StemCell institutional review board (IRB). No preference was given with regard to sex, ethnicity or race. Use of de-identified cells is considered exempt human subjects research and is approved by the UCSF IRB. T cells were isolated using the EasySep Human T cell isolation kit (StemCell Technologies, 100-0695) according to manufacturer’s instructions. Immediately after isolation, T cells were used directly for in vitro experiments. All T cells were cultured in complete X-VIVO 15 consisting of X-VIVO 15 (Lonza Bioscience, 04-418Q) supplemented with 5% FBS (R&D systems), 4 mM *N*-acetyl-cysteine (RPI, A10040) and 55 μM 2-mercaptoethanol (Gibco, 21985023). Pan CD3^+^ T cells were activated with anti-CD3/anti-CD28 Dynabeads (Gibco, 40203D) at a 1:1 bead-to-cell ratio in the presence of 500 IU ml^−1^ IL-2. Two days after stimulation, T cells were magnetically de-beaded and taken up in P3 buffer with supplement (Lonza Bioscience, V4SP-3096) at 37.5 × 10^6^ cells per ml. Next, 1.5 μg PEmax or PE7 mRNA mixed with 50 pmole synthetic pegRNA (Integrated DNA Technologies; Supplementary Table 8) was added per 20 µl cells, not exceeding 25 µl total volume per reaction. Cells were subsequently electroporated using a Lonza 4D Nucleofector with program DS-137. Immediately after electroporation, 80 µl warm complete X-VIVO15 was added to each electroporation well, and cells were incubated for 30 min in a 5% CO_2_ incubator at 37 °C followed by distribution of each electroporation reaction into 3 wells of a 96-well round-bottom plate. Each well was brought to 200 µl complete X-VIVO 15 and 200 IU ml^–1^ IL-2. Cells were subcultured and expanded through the addition of fresh medium and IL-2 every 2–3 days. Four days after electroporation, approximately 5 × 10^5^ cells were spun down at 500*g* for 5 min, and gDNA was extracted using a DNeasy Blood & Tissue kit (Qiagen, 69506) per the manufacturer’s instructions with an elution volume of 100 µl. To assess editing efficiency, PCR was performed with 25 µl of eluted gDNA per sample in a 100 µl PCR reaction with KAPA HiFi HotStart ReadyMix (Roche, 09420398001) with the following cycling conditions: 95 °C for 3 min, 28 cycles of (98 °C for 20 s, 63 °C for 15 s, and 72 °C for 60 s), followed by 72 °C for 2 min. PCR products were purified by SPRIselect (Beckman Coulter, B23317) and 2 µl eluted product was used for 8 cycles of additional PCR with KAPA HiFi HotStart ReadyMix to add Illumina sequencing adapters and indices. The final PCR products were purified by SPRIselect, quantified using a Qubit 1× dsDNA High Sensitivity assay kit (Invitrogen, Q33230), equimolarly pooled and sequenced using a MiSeq Reagent kit v2 300 cycles (Illumina, MS-102-2002) with 300 cycles for the R1 read, 8 cycles for the i7 index read and 8 cycles for the i5 index read. Sequencing data were demultiplexed using BaseSpace and analysed using CRISPResso2 (2.2.11).

### HSPC isolation, culture and prime editing

mRNA in vitro transcription template plasmids for HSPC experiments were constructed by cloning PEmax and PE7 into a previously described vector^63^. mRNA was generated using a HiScribe T7 High Yield RNA Synthesis kit (New England Biolabs, E2040S) and BbsI linearized plasmids as templates with UTP fully replaced by N^1^-methylpseudouridine-5′-triphosphate (TriLink Biotechnologies, N-1081) and co-transcriptional capping by CleanCap Reagent AG (TriLink Biotechnologies, N-7113). Following IVT, mRNA was purified using a Monarch RNA Cleanup kit (500 µg) (NEB, T2050S), eluted in IDTE pH 7.5 (Integrated DNA Technologies, 11-05-01-15) and quantified using a Qubit RNA High Sensitivity Assay kit (Invitrogen, Q32852). Synthetic pegRNAs and an epegRNA were ordered as Custom Alt-R gRNA from Integrated DNA Technologies (Supplementary Table 8) and resuspended at 200 µM in IDTE pH 7.5. Cryopreserved human CD34^+^ HSPCs from mobilized peripheral blood of de-identified healthy donors were obtained from the Fred Hutchinson Cancer Research Center (Seattle, Washington). The CD34^+^ HSPCs used in this study were de-identified and research use consent had been previously obtained. As the de-identified human specimens were not collected specifically for this study and our study team could not access any subject identifiers linked to the specimens or data, the Boston Children’s Hospital IRB has determined this is not considered human-related research. CD34^+^ HSPCs were cultured with X-Vivo-15 medium supplemented with 100 ng ml^−1^ human stem cell growth factor, 100 ng ml^−1^ human thrombopoietin and 100 ng ml^−1^ recombinant human FMS-like tyrosine kinase 3 ligand. CD34^+^ HSPCs were thawed and cultured for 24 h in the presence of cytokines before nucleofection. Overall, 2.5 × 10^5^ CD34^+^ HSPCs were electroporated using a P3 Primary Cell X kit S (Lonza Bioscience, V4SP-3096) according to the manufacturer’s recommendations with 2,000 ng PEmax or PE7 mRNA and 200 pmole synthetic pegRNA or epegRNA using pulse code DS-130. gDNA was collected 3 days after nucleofection using QuickExtract DNA Extraction solution (LGC Biosearch Technologies, QE09050) following the manufacturer’s recommendations. Prime editing outcomes were quantified by amplicon sequencing and CRISPResso2 (2.2.11) analysis as described above.

### Statistics and reproducibility

CRISPRi screens were performed in independent biological duplicate. Sample sizes (*n*) for all other experiments and analyses are defined in the appropriate main or extended data figure legend and experiments were performed as described therein, with the following exceptions. Results in Fig. 2a (and Extended Data Fig. 3d) are from western blotting performed once with specified cell lines. Results in Fig. 2f depict representative flow cytometry plots (*n* = 3 independent biological replicates). For all instances of *n* ≤ 10, data points were plotted individually (in relevant or associated figure panel) and/or data are provided in Supplementary Tables 1–3 and 7 or raw data have been made publicly available, except for gene-level phenotypes of our PE4 and PE5 genome-scale CRISPRi screens, from which no significant hits were identified. Select comparisons between editing conditions are indicated in Figs. 1e, 2b,c, 3d, 4b,c,f, 5a,d,f and Extended Data Figs. 3a,b,h, 4a,b, 5c–e, 9a,b,d, 10a and 11d. *P* values for these comparisons can be found in the associated figure panels or in Supplementary Table 7.

### Reporting summary

Further information on research design is available in the Nature Portfolio Reporting Summary linked to this article.

## Online content

Any methods, additional references, Nature Portfolio reporting summaries, source data, extended data, supplementary information, acknowledgements, peer review information; details of author contributions and competing interests; and statements of data and code availability are available at 10.1038/s41586-024-07259-6.

## Supplementary information

Supplementary Information

Reporting Summary

Supplementary Tables Supplementary Tables 1–10.

## Data Availability

GRCh38.p13 (GCA_000001405.28, PRJNA31257) from Ensembl release 107 used for small RNA sequencing analysis is available at http://ftp.ensembl.org/pub/release-107/fasta/homo_sapiens/dna/Homo_sapiens.GRCh38.dna.primary_assembly.fa.gz. GENCODE gene annotation release 43 used for small RNA sequencing analysis is available at https://ftp.ebi.ac.uk/pub/databases/gencode/Gencode_human/release_43/gencode.v43.primary_assembly.annotation.gff3.gz. GRCh38.p13 (GCA_000001405.28, PRJNA31257) from Ensembl release 100 used for RNA sequencing is available at https://ftp.ensembl.org/pub/release-100/fasta/homo_sapiens/dna/Homo_sapiens.GRCh38.dna.primary_assembly.fa.gz. High-throughput sequencing data of primary human T cell experiments have been deposited into the Gene Expression Omnibus (GEO) database (identifier GSE255003) and the NCBI Sequence Read Archive database under accession PRJNA1073019. High-throughput sequencing data of primary human HSPC experiments have been deposited at the NCBI Sequence Read Archive database under accession PRJNA1071146. All other high-throughput sequencing data have been deposited into the GEO (identifier GSE253424) and the NCBI Sequence Read Archive database under accession PRJNA1065772.

## Code availability

The code for small RNA sequencing analysis is available at Zenodo (10.5281/zenodo.10553303)^50^ or GitHub (https://github.com/Princeton-LSI-ResearchComputing/PE-small-RNA-seq-analysis)^51^. The code for RNA sequencing analysis is available at Zenodo (10.5281/zenodo.10553340)^59^ or at GitHub (https://github.com/Princeton-LSI-ResearchComputing/PE-mRNA-seq-diffexp)^60^.
