# Supplementary material for: Improving prime editing with an endogenous small RNA-binding protein
Source: Nature. 2024 Apr 3;628(8008):639–47. doi: 10.1038/s41586-024-07259-6 (PMC11023932; doi:10.1038/s41586-024-07259-6)
Supplement: Supplementary file 1 — Supplementary Information [file 41586_2024_7259_MOESM1_ESM.docx]

**Improving prime editing with an endogenous small RNA-binding protein**

Jun Yan^1^, Paul Oyler-Castrillo^2^, Purnima Ravisankar^2,18^, Carl C. Ward^3^, Sébastien Levesque^4,5,6,7,8^, Yangwode Jing^9^, Danny Simpson^2^, Anqi Zhao^1^, Hui Li^1^, Weihao Yan^1^, Laine Goudy^3,10,11^, Ralf Schmidt^3,12^, Sabrina C. Solley^1^, Luke A. Gilbert^11,13,14,15^, Michelle Chan^1,2^, Daniel E. Bauer^4,5,6,7,8^, Alexander Marson^3,15,16,17^, Lance R. Parsons^2^ & Britt Adamson^1,2,*^

^1^Department of Molecular Biology, Princeton University, Princeton, NJ, USA.

^2^Lewis–Sigler Institute for Integrative Genomics, Princeton University, Princeton, NJ, USA.

^3^Gladstone–UCSF Institute of Genomic Immunology, San Francisco, CA, USA.

^4^Division of Hematology/Oncology, Boston Children’s Hospital, Boston, MA, USA.

^5^Department of Pediatric Oncology, Dana–Farber Cancer Institute, Boston, MA, USA.

^6^Harvard Stem Cell Institute, Cambridge, MA, USA.

^7^Broad Institute, Cambridge, MA, USA.

^8^Department of Pediatrics, Harvard Medical School, Boston, MA, USA.

^9^Department of Chemistry, Princeton University, Princeton, NJ, USA.

^10^Biomedical Sciences Graduate Program, University of California, San Francisco, San Francisco, CA, USA.

^11^Arc Institute, Palo Alto, CA, USA.

^12^Department of Laboratory Medicine, Medical University of Vienna, Vienna, Austria.

^13^Department of Urology, University of California, San Francisco, San Francisco, CA, USA.

^14^Helen Diller Family Comprehensive Cancer Center, University of California, San Francisco, San Francisco, CA, USA.

^15^Innovative Genomics Institute, University of California, Berkeley, Berkeley, CA, USA.

^16^Department of Medicine, University of California, San Francisco, San Francisco, CA, USA.

^17^Parker Institute for Cancer Immunotherapy, San Francisco, CA, USA.

^18^Present address: Immunology and Microbial Pathogenesis Program, Weill Cornell Graduate School of Medical Sciences, New York, NY, USA.

*Correspondence: badamson@princeton.edu (B.A.)

**Supplementary Information**

[Supplementary Discussion 3](#_Toc159346731)

[Supplementary Tables 5](#_Toc159346732)

[Supplementary Figure 1. 6](#_Toc159346733)

[Supplementary Figure 2. 7](#_Toc159346734)

[Supplementary Figure 3. 8](#_Toc159346735)

[Supplementary Figure 4. 9](#_Toc159346736)

[Supplementary Figure 5. 10](#_Toc159346737)

[Supplementary Figure 6. 11](#_Toc159346738)

[Supplementary Figure 7. 12](#_Toc159346739)

[Supplementary Figure 8. 13](#_Toc159346740)

[Supplementary References 14](#_Toc159346741)

# Supplementary Discussion

**Loss of La variably destabilizes (e)pegRNAs**

To explore the possibility that La impacts the stability and integrity of (e)pegRNAs, we performed small RNA sequencing to characterize eleven transiently expressed (e)pegRNAs targeting seven genomic loci from K562 PEmax parental and La-ko4 cells (Extended Data Fig. 6 and 7, Methods). We defined RNA fragments from paired-end sequencing reads (Extended Data Fig. 6a, b, Methods), with sequences between non-overlapping paired-end reads inferred from the alignments. We then categorized fragments uniquely mapped to individual (e)pegRNAs (97.4 ± 0.7% of total (e)pegRNA aligning fragments) into three mutually exclusive bins (Extended Data Fig. 6c): (1) Our ‘*cis*-active’ bin included fragments with ≥15 nt of spacer and a defined region of the sgRNA scaffold. These fragments represent (e)pegRNAs with the minimal sequence for binding to Cas9 nickase and efficient target engagement^1,2,3,4,5^. (2) Our ‘*trans*-active’ bin received any remaining fragments containing at least the edit-encoding nucleotide and the first 5′ nucleotide of the primer binding site (PBS). Given reports that untethered templates can enable efficient prime editing^6,7^, we reasoned that these fragments are necessary but not sufficient for templating prime editing in *trans*. (3) Remaining fragments were called ‘inactive’, including those ending at cryptic terminator sequences^8^.

Expression analysis^9^ revealed that the abundance of many *cis*-active, *trans*-active, and premature termination fragments were reduced in La-ko4 cells relative to parental K562 PEmax cells one and two days post transfection (Extended Data Fig. 6d), which suggests a role for La in stabilizing Pol III-transcribed (e)pegRNAs. To further explore potential stability and integrity phenotypes, we counted the number of fragments (coverage) at each nucleotide position within each bin for each (e)pegRNA and normalized those counts in two ways: either to the number of fragments from the same sample assigned to human RNA (absolute coverage) or to the number of fragments within the corresponding bin (relative coverage). Examining these coverages revealed two major observations (Extended Data Fig. 6e, f, 7a-c):

1. Independent of the presence or absence of La, *cis*-active fragments typically contained the majority of their spacer and scaffold regions (labeled ‘sgRNA sequence’ in figures) but, as early as one day post transfection, lacked large portions of their 3′ extensions. While similar to published observations of pegRNA 3′ extension degradation in HEK293T cells^6,10,11^, our data revealed steep and reproducible decreases in coverages at particular positions suggesting stepwise or heterogeneous end processing. Additionally, when 3′ extensions were completely lost, the last 1-3 nucleotides of sgRNA sequence were also often missing according to coverage plots, consistent with a previous observation that the stem loop at the 3′ end of sgRNAs makes very few contacts with Cas9^5,12^.
2. Loss of La exacerbated truncation of 3′ extensions on *cis*-active (e)pegRNA fragments according to relative coverages. To quantify this observation, we calculated and compared a metric of (e)pegRNA integrity across K562 PEmax parental and La-ko4 cells (Extended Data Fig. 7d-f) and found that loss of La significantly reduced the percentage of *cis*-active fragments containing the edit-encoding nucleotide one and two days post transfection for eight of the eleven (e)pegRNAs (*P* ≤ 0.05).

These results suggest that loss of La destabilizes (e)pegRNAs and renders their 3′ ends particularly unstable.

To supplement these analyses, we performed another small RNA sequencing experiment using a pegRNA and epegRNA pair (*Mus* DNMT1 +6 G to C) with no obvious genomic target in human cells^13^ (Extended Data Fig 8a, b). The rationale for this experiment was as follows: Because prime editing generates RNA-DNA hybrids during editing^14^, cellular RNases (RNase H1/H2) or the intrinsic RNase H activity of the MMLV reverse transcriptase (MMLV-RT) in PEmax could be responsible for some of the 3′ end loss observed in our analyses of targeting (e)pegRNAs. By evaluating a non-targeting pegRNA and epegRNA pair with small RNA sequencing, we reasoned that we could evaluate (e)pegRNA stability and integrity without RNase H-mediated degradation. Results from this experiment showed that, unlike targeting (e)pegRNAs, most *cis*-active fragments from the non-targeting (e)pegRNAs retained 3′ extensions in parental K562 PEmax cells one day post transfection, and truncation patterns were nearly bimodal (Extended Data Fig. 8c). Similar to the targeting (e)pegRNAs, though, this non-targeting pair demonstrated preferential loss of 3′ extensions in La-ko4 cells compared to parental K562 PEmax cells (8.1% and 12.3% decrease in percentage of *cis*-active fragments with the edit-encoding nucleotide for the non-targeting pegRNA on day 1 and 2, respectively, and 76.7% and 79.4% decrease for the non-targeting epegRNA, Extended Data Fig. 8c-e). These results therefore also support a protective interaction between La and the 3′ ends of expressed (e)pegRNAs.

However, for the non-targeting pegRNA, truncation of *cis*-active fragments was only subtly exacerbated in La-ko4 cells and absolute coverages of those fragments were similar between cell lines one day post transfection. These results suggest minimal instability or truncation for the pegRNA at that timepoint, but in a separate experiment, we observed strongly reduced intended prime editing efficiencies with an exogenously provided target in La-ko4 cells one day post transfection (Extended Data Fig. 8f). We therefore reason that the quantitative relationship between (e)pegRNA integrity and editing may be complex (*i.e.*, nonlinear) and/or that protecting (e)pegRNAs in the absence of a target represents only part of La’s role in prime editing (Fig. 3f). We therefore must allow for the possibility that La also promotes other steps in prime editing, such as nuclear retention of (e)pegRNAs or effector complex formation^10,11,15^. Alternatively, La may impact (e)pegRNA stability and integrity in multiple ways, for example by offering protection from cellular exonucleases as well as possible modes of target-dependent degradation (possibly by RNase H).

**Expanded description of PE7 off-target analysis**

We also evaluated off-target editing in U2OS cells by sequencing several common Cas9 off-target sites^16^ associated with four genomic loci previously used to evaluate prime editing specificity^10,14,17^ (Extended Data Fig. 9d). Frequencies of off-target editing at these thirteen sites were very low (median 0.053% and 0.065% of aligned sequencing reads for PEmax and PE7, respectively), and compared to PEmax, PE7 increased off-target editing at only two sites, suggesting that, while PE7 generally boosts prime editing, few off-target sites are susceptible to that effect.

# Supplementary Tables

**Supplementary Table 1.** sgRNA-level phenotypes from genome-scale CRISPRi screens in this study.

**Supplementary Table 2.** Gene-level phenotypes from genome-scale CRISPRi screen with the FACS reporter using the PE3 approach in this study.

**Supplementary Table 3.** Gene-level phenotypes from genome-scale CRISPRi screen with the MCS reporter using the PE3 approach in this study**.**

**Supplementary Table 4.** Sequences of pegRNAs and sgRNAs used throughout the study.

**Supplementary Table 5.** Sequences of fusion prime editor proteins used throughout the study.

**Supplementary Table 6.** Sequences of primers used throughout the study.

**Supplementary Table 7.** Flow cytometry analyses, additional statistical test results, individual data points from experiments and CRISPResso2 analysis window sizes.

**Supplementary Table 8.** Synthetic guide RNAs used throughout the study.

**Supplementary Table 9.** Sequences of amplicons analyzed with high-throughput sequencing.

**Supplementary Table 10.** RNA sequencing results. Adjusted *P*-values calculated by DESeq2 using the two-tailed Wald test with Benjamini-Hochberg correction.

**
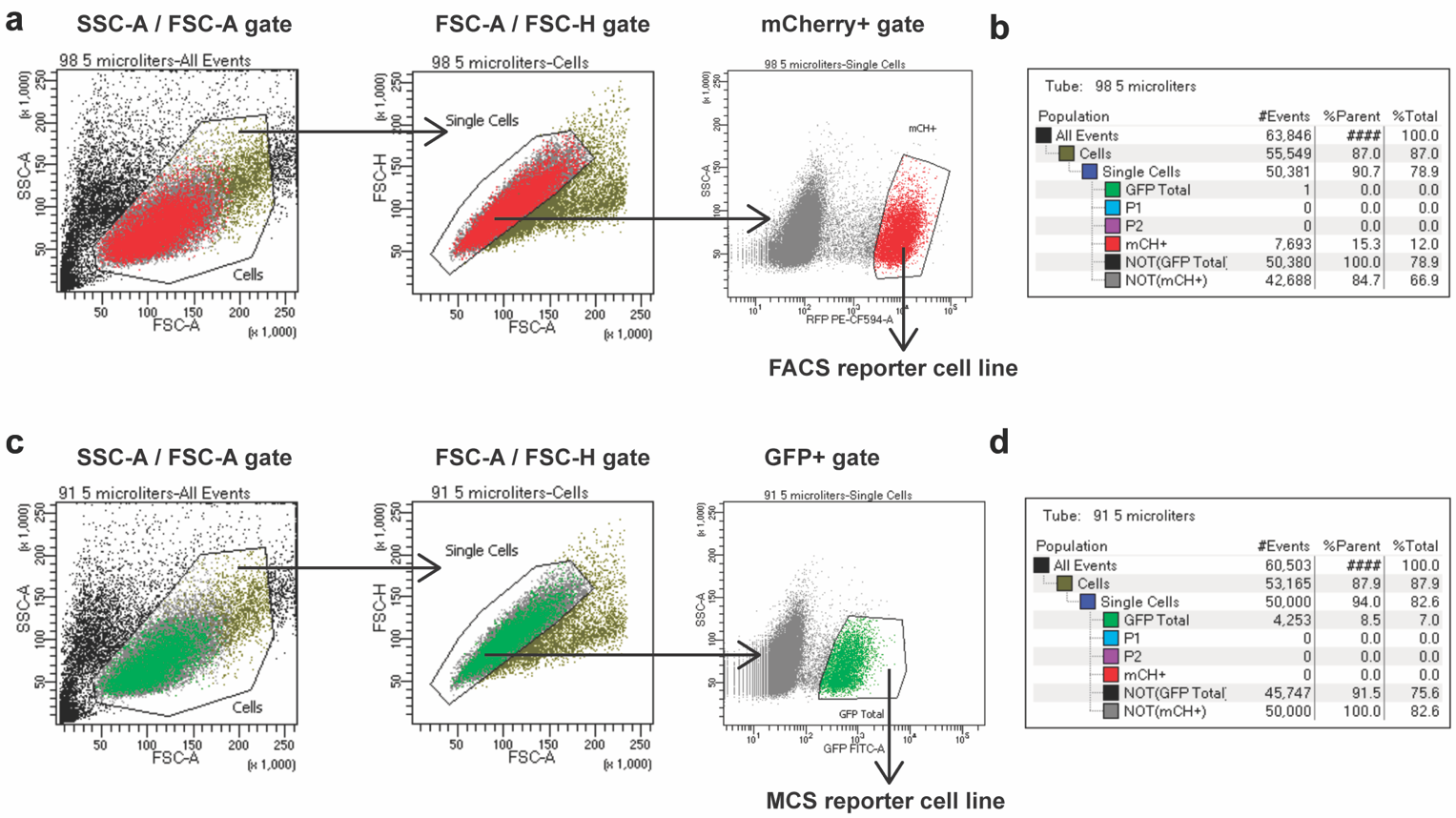
**

# Supplementary Figure 1.

**Supplementary Figure 1.** Gating strategies used for construction of FACS and MCS reporter cell lines. **a,** For the FACS reporter line, cells were gated on FSC-A/SSC-A to exclude debris (left, black polygon), then on FSC-A/FSC-H to select single cells (middle, black polygon), and then on mCherry to select those cells expressing the FACS reporter-associated marker gene. Here, we depict images from analysis of the FACS reporter line sort. **b,** Event numbers and percentages from data in a. **c,** For the MCS reporter line, cells were gated on FSC-A/SSC-A to exclude debris (left, black polygon), then on FSC-A/FSC-H to select single cells (middle, black polygon), and then on GFP to select those cells expressing the MCS reporter-associated marker gene. Here, we depict images from analysis of the MCS reporter line sort. **d,** Event numbers and percentages from data in c. Cell numbers for all samples provided in Supplementary Table 7. Analyses performed with BD FACSDiva (8.0.1).


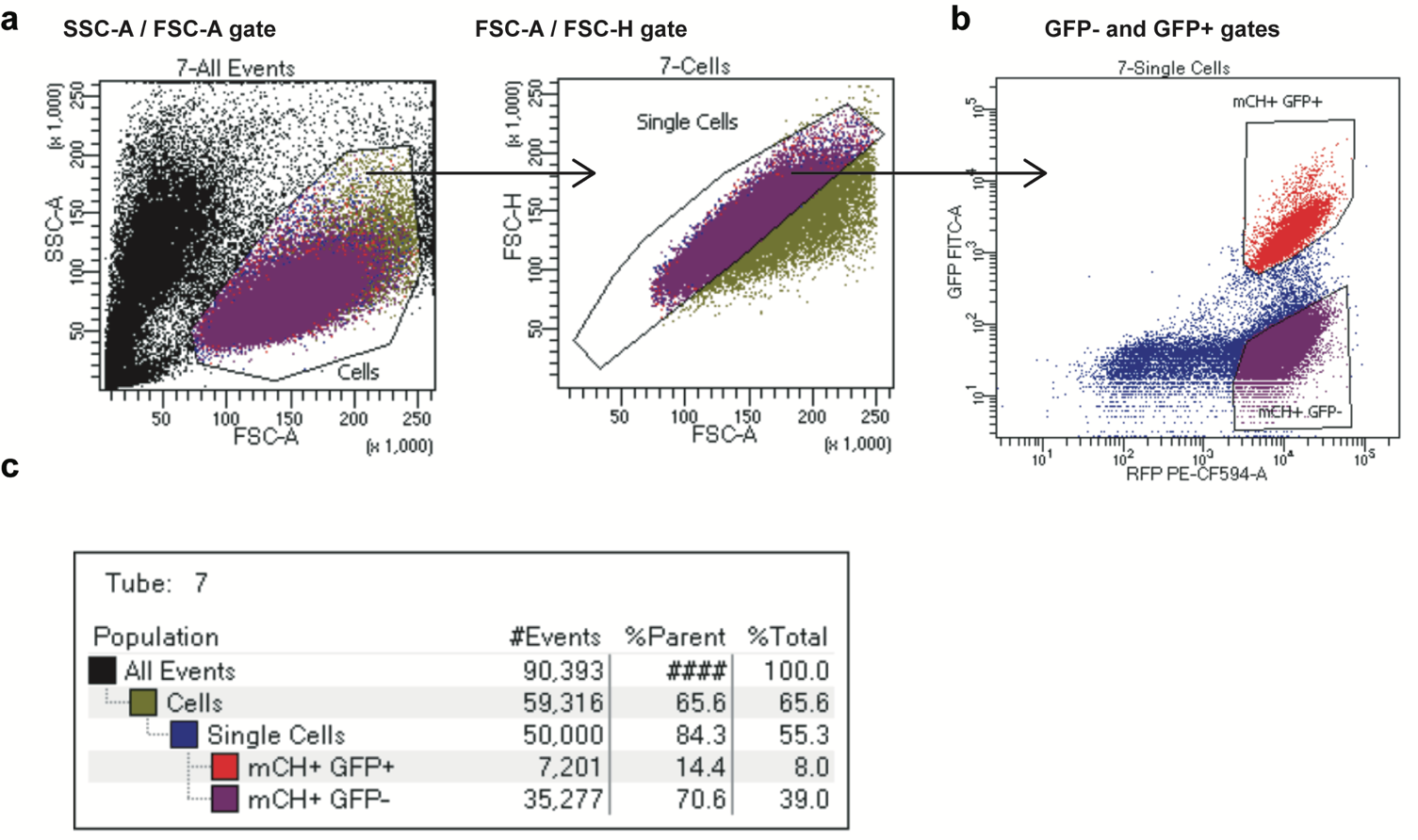


# Supplementary Figure 2.

**Supplementary Figure 2.** Gating strategy used for FACS reporter validation experiment presented in Extended Data Fig. 1f. **a,** Cells were first gated on FSC-A/SSC-A to exclude debris (left, black polygon) and then on FSC-A/FSC-H to select single cells (right, black polygon). **b**, Final gate used to sort mCherry+, GFP+ and mCherry+, GFP- cells. mCherry is the FACS reporter marker and GFP expression is indicative of successful prime editing. **c,** Event numbers and percentages from data in a and b. Here, we depict images from analysis of cells edited with +7 GG to CA using the PE3 approach. Cell numbers for all samples provided in Supplementary Table 7. Analyses performed with BD FACSDiva (8.0.1).

**
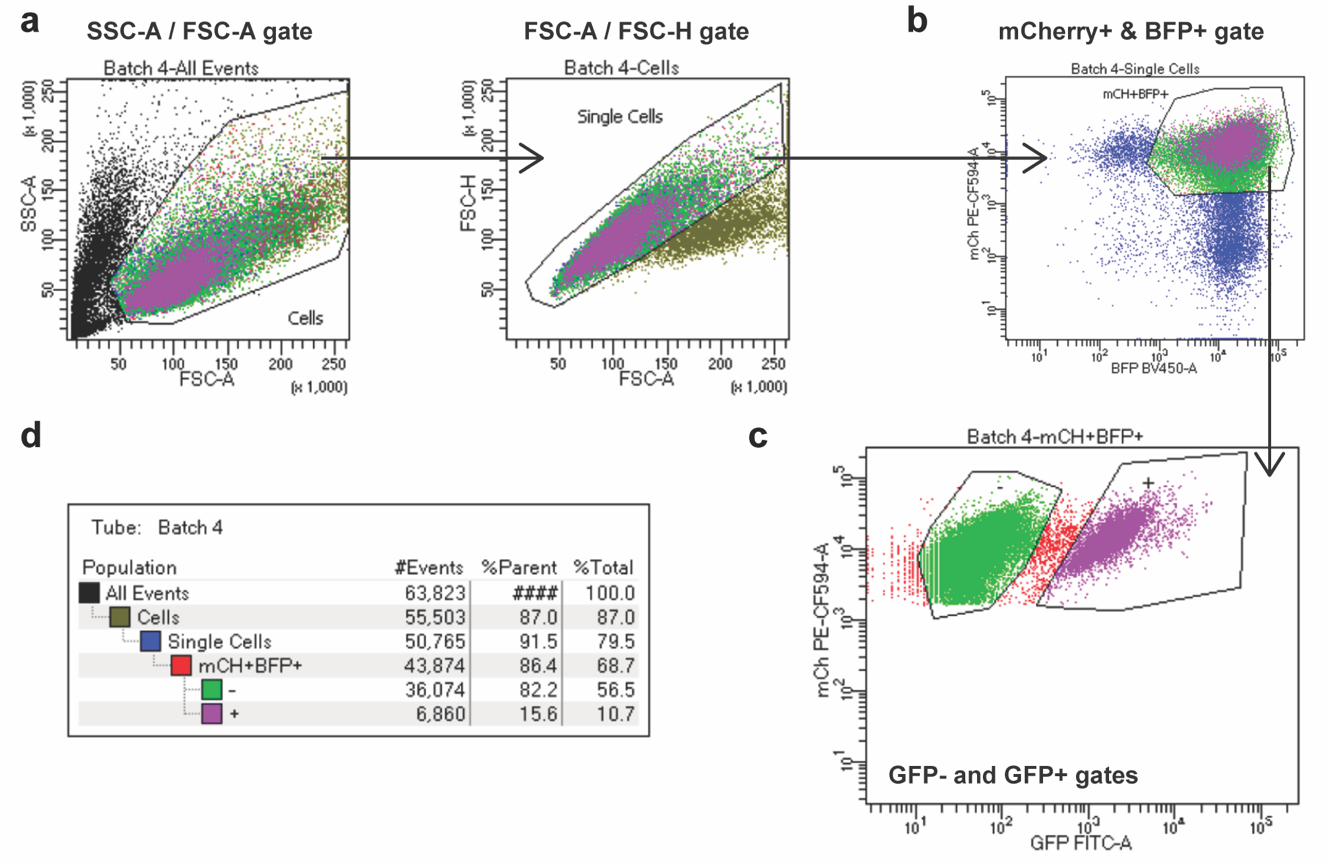
**

# Supplementary Figure 3.

**Supplementary Figure 3.** Example gating strategy used for FACS-based genome-scale CRISPRi screen (associated data presented in Fig. 1c and Extended Data Fig. 1i, j, 2a-c, f). **a,** Cells were first gated on FSC-A/SSC-A to exclude debris (left, black polygon) and then on FSC-A/FSC-H to select single cells (right, black polygon). **b**, Example third gate (black polygon) used to select cells expressing mCherry (indicative of FACS reporter integration) and BFP (indicative of CRISPRi sgRNA expression). **c,** Example final gate used to sort GFP+ and GFP- cells. GFP expression is indicative of successful prime editing. **d,** Event numbers and percentages from data in a-c. Here, we depict images from analysis of one batch sort of replicate 1 of the screen. GFP profiles for each batch sort of both replicates are included in Extended Data Fig. 1h. Cell numbers from all batch sorts are provided in Supplementary Table 7. Analyses performed with BD FACSDiva (8.0.1).

**
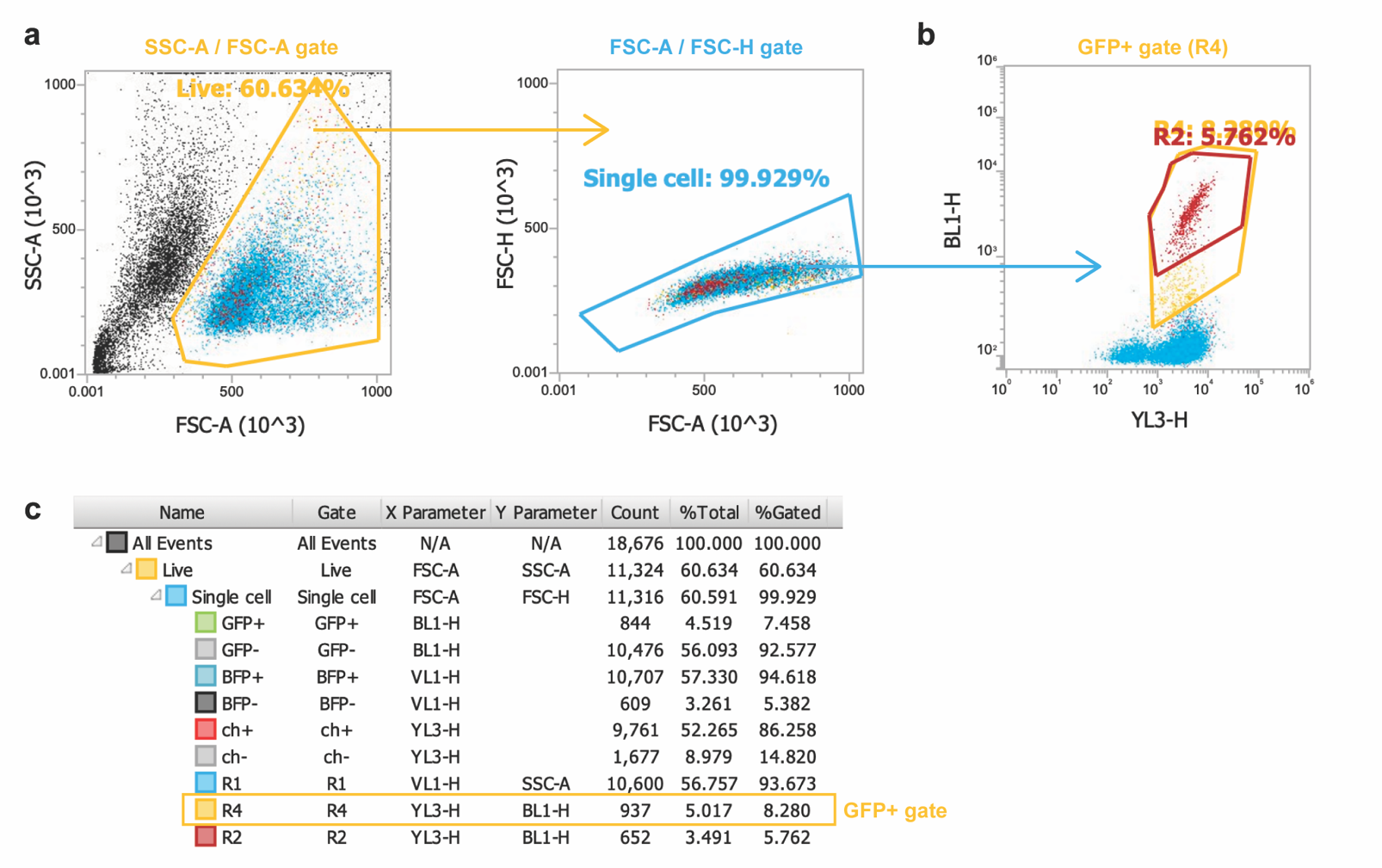
**

# Supplementary Figure 4.

**Supplementary Figure 4.** Gating strategy used for analysis presented in Extended Data Fig. 3a. **a,** Cells were first gated on FSC-A/SSC-A to exclude debris (left, yellow polygon) and then on FSC-A/FSC-H to select single cells (right, blue polygon). **b,** Final gate (yellow polygon) used for quantification of GFP. **c,** Event numbers and percentages from data in a and b. Here, we depict images from analysis of one NT_2, epegRNA replicate. Cell numbers and percentages from all samples are depicted in Extended Data Fig. 3a and provided in Supplementary Table 7. Analysis performed with Attune Cytometric Software (5.2.0).

**
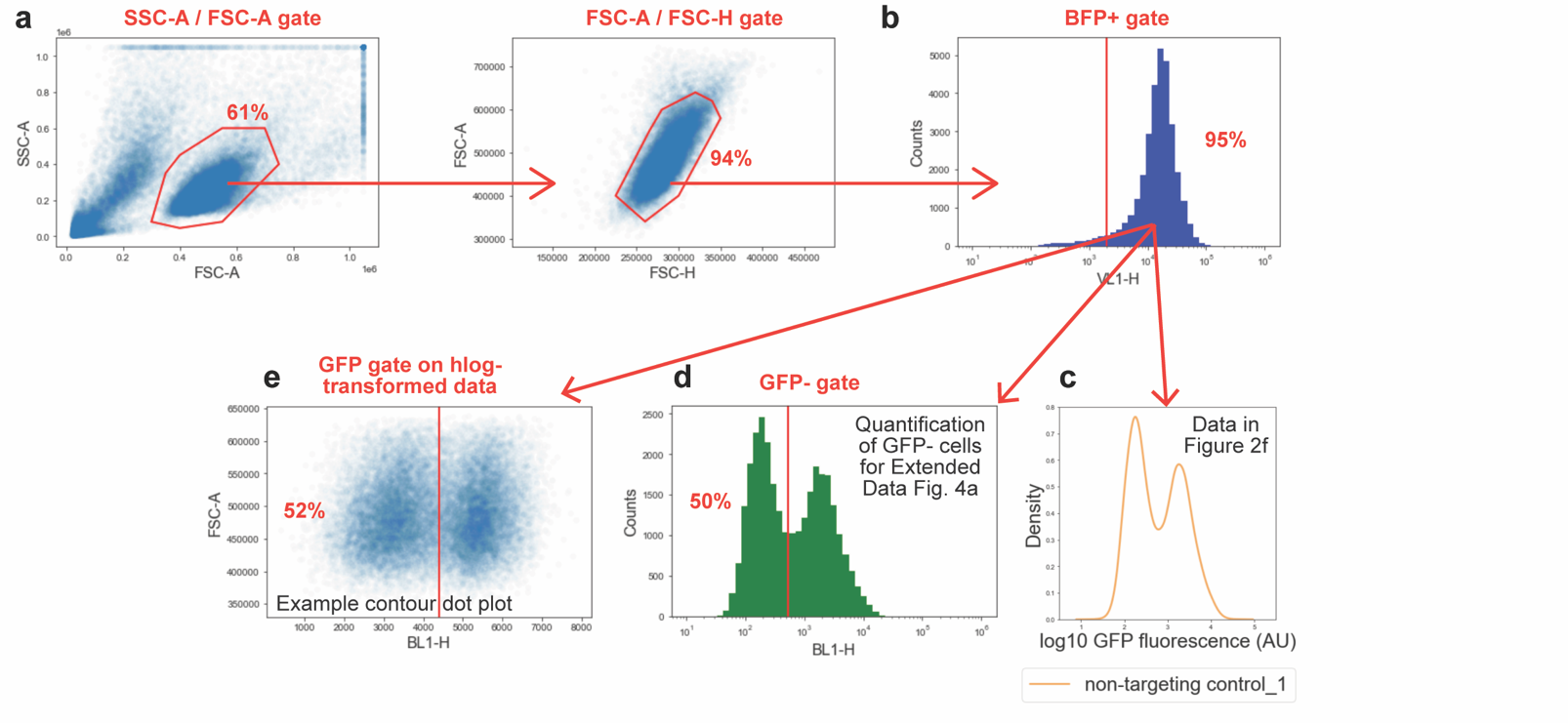
**

# Supplementary Figure 5.

**Supplementary Figure 5.** Example gating strategy for analyses of flow cytometry data presented in Fig. 2f and Extended Data Fig. 1d, e, h, 3g, 4a, demonstrated using data presented in Fig. 2f and Extended Data Fig. 4a. **a,** Cells were first gated on FSC-A/SSC-A to exclude debris (left, red polygon) and then gated on FSC-A/FSC-H to select single cells (right, red polygon). **b,** Example third gate (red line) used to select cells according to transgene marker expression (mCherry and/or BFP). This gate or similar was used or omitted as appropriate for each experiment. Here, we depict a gate used to select BFP+ cells (indicative of CRISPRi sgRNA expression) from the cell population selected in a. **c,** Example kernel density estimate plot (analogous to a histogram) used for presentation of flow cytometry data in Fig. 2f, Extended Data Fig. 1d, e, h, and 3g. Here, we depict the distribution of GFP expression from the cell population selected in b. **d,** Example gating strategy used for quantification of GFP+/GFP- cells. Histogram depicts the cell population selected in b. **e,** Example contour dot plot of cell population selected in b with an independent gate separating GFP+/GFP- cells. Gates depicted here were used for analysis of data presented in Fig. 2f and Extended Data Fig. 4a. Similar but not exact gates were used for data depicted in Extended Data Fig. 1d, e, h, and 3g. Cell numbers and percentages associated with each of these figures are provided in Supplementary Table 7. Analyses of flow cytometry data were performed using FlowCytometryTools^18^ (0.5.1, https://github.com/eyurtsev/FlowCytometryTools).

**
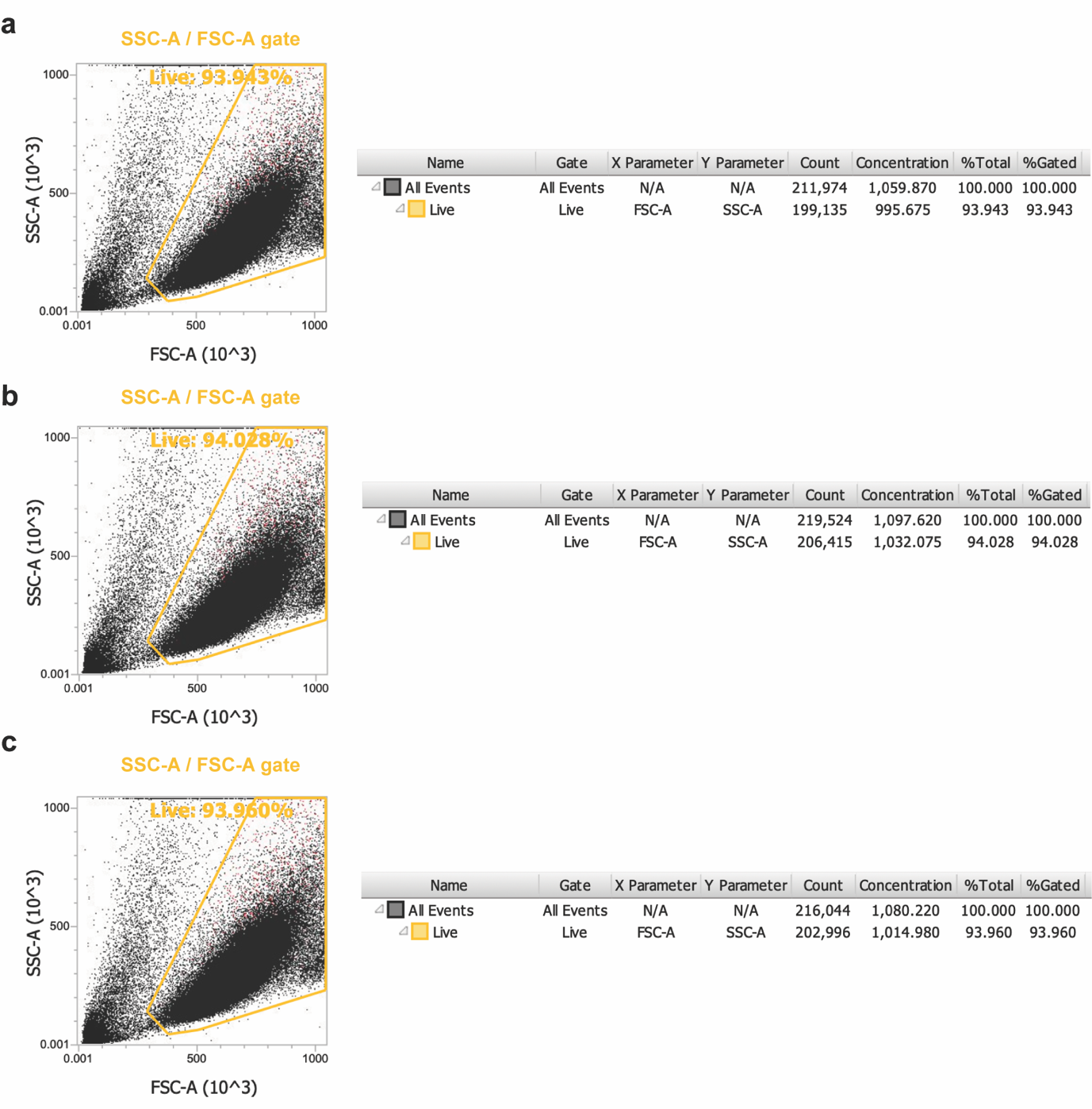
**

# Supplementary Figure 6.

**Supplementary Figure 6.** Flow cytometry gating strategy used to determine cell numbers for analysis presented in Extended Data Fig. 3f. **a,** Left: To quantify viable cells, events were gated on FSC-A/SSC-A (left, yellow polygon). Right: Event numbers and percentages from gate depicted on the left. **b**, Same as a, but for a second replicate. **c,** Same as a, but for a third replicate. Here, we depict images from n=3 independent biological replicates of parental K562 PEmax cells. Cell numbers from all samples provided in Supplementary Table 7. Analysis performed with Attune Cytometric Software (5.2.0).

**
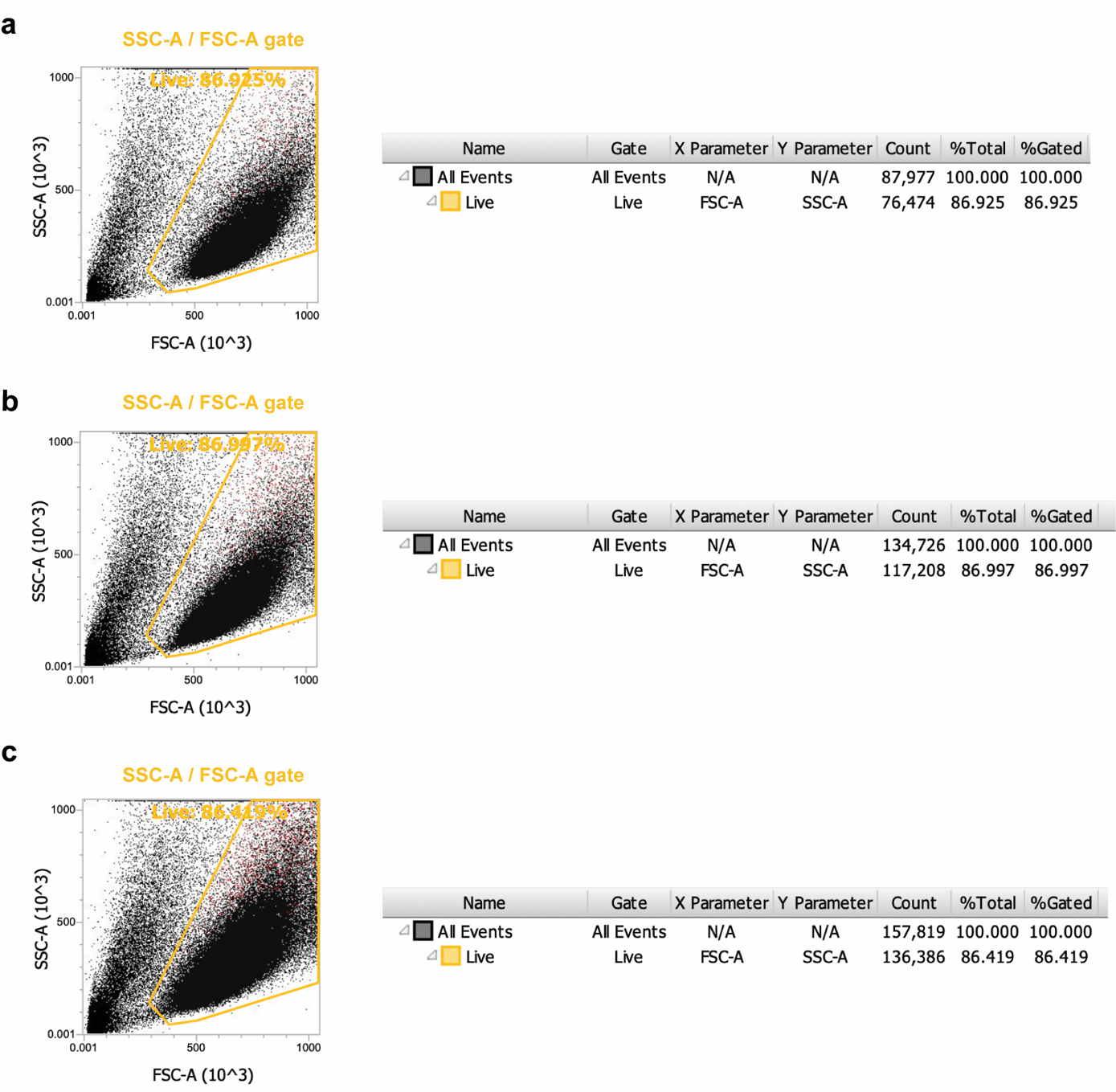
**

# Supplementary Figure 7.

**Supplementary Figure 7.** Flow cytometry gating strategy used to determine cell numbers for analyses presented in Extended Data Fig. 10b and c. **a,** Left: To quantify viable cells, events were gated on FSC-A/SSC-A (left, yellow polygon). Data from day 1. Right: Event numbers and percentages from gate depicted on the left. **b**, Same as a, but from day 2. **c,** Same as a, but from day 3. Here, we depict images from analysis of one replicate (of four total) for the HEK3 +1 T to A, PEmax condition. Percentages from all samples depicted in Extended Data Fig. 10b. Cell numbers from all samples provided in Supplementary Table 7. Analyses performed with Attune Cytometric Software (5.2.0).

**
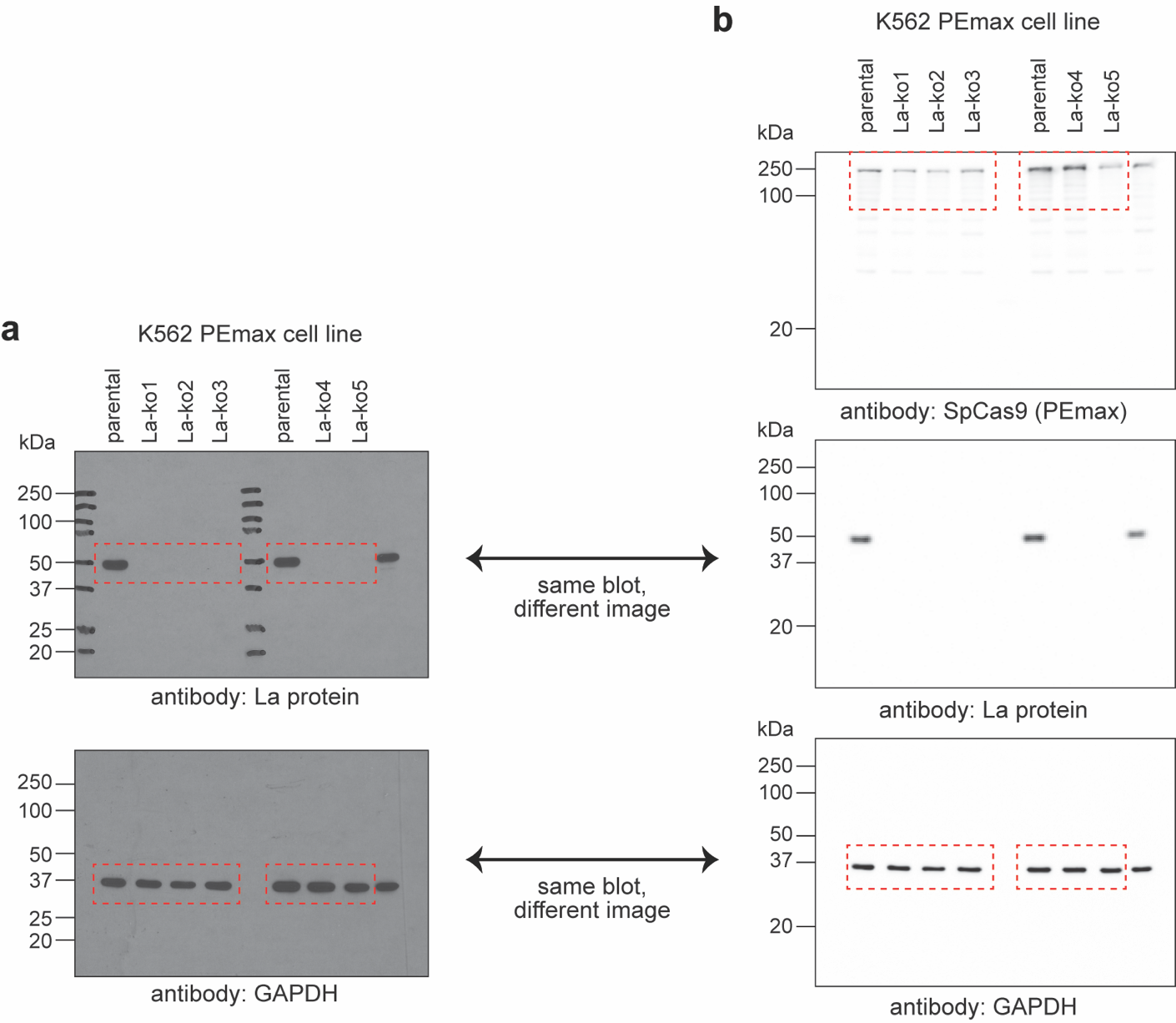
**

# Supplementary Figure 8.

**Supplementary Figure 8.** Full images of western blot analysis presented in Fig. 2a (a) and Extended Data Fig. 3d (b), with dotted outlines showing the excerpted portions. All images are from the same membrane, but results in a were developed with films (SpCas9 not imaged with this technique), while results in b were taken with Azure Biosystems 600 (resulting La image was overly pixelated when examined post imaging). For figure generation, we used the clearest result for La (a) and the only image of SpCas9 (b) and included the similarly imaged GAPDH results (a and b, respectively) as corresponding loading control.

# Supplementary References

1. Jinek, M., Chylinski, K., Fonfara, I., Hauer, M., Doudna, J. A. & Charpentier, E. A programmable dual-RNA-guided DNA endonuclease in adaptive bacterial immunity. *Science* **337,** 816–821 (2012).
2. Cong, L., Ran, F. A., Cox, D., Lin, S., Barretto, R., Habib, N., Hsu, P. D., Wu, X., Jiang, W., Marraffini, L. A. & Zhang, F. Multiplex genome engineering using CRISPR/Cas systems. *Science* **339,** 819–823 (2013).
3. Jinek, M., East, A., Cheng, A., Lin, S., Ma, E. & Doudna, J. RNA-programmed genome editing in human cells. *Elife* **2,** e00471 (2013).
4. Fu, Y., Sander, J. D., Reyon, D., Cascio, V. M. & Joung, J. K. Improving CRISPR-Cas nuclease specificity using truncated guide RNAs. *Nat. Biotechnol.* **32,** 279–284 (2014).
5. Nishimasu, H., Ran, F. A., Hsu, P. D., Konermann, S., Shehata, S. I., Dohmae, N., Ishitani, R., Zhang, F. & Nureki, O. Crystal structure of Cas9 in complex with guide RNA and target DNA. *Cell* **156,** 935–949 (2014).
6. Liu, B., Dong, X., Cheng, H., Zheng, C., Chen, Z., Rodríguez, T. C., Liang, S.-Q., Xue, W. & Sontheimer, E. J. A split prime editor with untethered reverse transcriptase and circular RNA template. *Nat. Biotechnol.* **40,** 1388–1393 (2022).
7. Feng, Y., Liu, S., Mo, Q., Liu, P., Xiao, X. & Ma, H. Enhancing prime editing efficiency and flexibility with tethered and split pegRNAs. *Protein Cell* (2022). doi:10.1093/procel/pwac014
8. Chen, B., Gilbert, L. A., Cimini, B. A., Schnitzbauer, J., Zhang, W., Li, G.-W., Park, J., Blackburn, E. H., Weissman, J. S., Qi, L. S. & Huang, B. Dynamic imaging of genomic loci in living human cells by an optimized CRISPR/Cas system. *Cell* **155,** 1479–1491 (2013).
9. Love, M. I., Huber, W. & Anders, S. Moderated estimation of fold change and dispersion for RNA-seq data with DESeq2. *Genome Biol.* **15,** 550 (2014).
10. Nelson, J. W., Randolph, P. B., Shen, S. P., Everette, K. A., Chen, P. J., Anzalone, A. V., An, M., Newby, G. A., Chen, J. C., Hsu, A. & Liu, D. R. Engineered pegRNAs improve prime editing efficiency. *Nat. Biotechnol.* **40,** 402–410 (2022).
11. Ponnienselvan, K., Liu, P., Nyalile, T., Oikemus, S., Maitland, S. A., Lawson, N. D., Luban, J. & Wolfe, S. A. Reducing the inherent auto-inhibitory interaction within the pegRNA enhances prime editing efficiency. *Nucleic Acids Res.* **51,** 6966–6980 (2023).
12. Jiang, F., Taylor, D. W., Chen, J. S., Kornfeld, J. E., Zhou, K., Thompson, A. J., Nogales, E. & Doudna, J. A. Structures of a CRISPR-Cas9 R-loop complex primed for DNA cleavage. *Science* **351,** 867–871 (2016).
13. Labun, K., Montague, T. G., Krause, M., Torres Cleuren, Y. N., Tjeldnes, H. & Valen, E. CHOPCHOP v3: expanding the CRISPR web toolbox beyond genome editing. *Nucleic Acids Res.* **47,** W171–W174 (2019).
14. Anzalone, A. V., Randolph, P. B., Davis, J. R., Sousa, A. A., Koblan, L. W., Levy, J. M., Chen, P. J., Wilson, C., Newby, G. A., Raguram, A. & Liu, D. R. Search-and-replace genome editing without double-strand breaks or donor DNA. *Nature* **576,** 149–157 (2019).
15. Wolin, S. L. & Cedervall, T. The La protein. *Annu. Rev. Biochem.* **71,** 375–403 (2002).
16. Tsai, S. Q., Zheng, Z., Nguyen, N. T., Liebers, M., Topkar, V. V., Thapar, V., Wyvekens, N., Khayter, C., Iafrate, A. J., Le, L. P., Aryee, M. J. & Joung, J. K. GUIDE-seq enables genome-wide profiling of off-target cleavage by CRISPR-Cas nucleases. *Nat. Biotechnol.* **33,** 187–197 (2015).
17. Chen, P. J., Hussmann, J. A., Yan, J., Knipping, F., Ravisankar, P., Chen, P.-F., Chen, C., Nelson, J. W., Newby, G. A., Sahin, M., Osborn, M. J., Weissman, J. S., Adamson, B. & Liu, D. R. Enhanced prime editing systems by manipulating cellular determinants of editing outcomes. *Cell* **184,** 5635–5652.e29 (2021).
18. Yurtsev, E. & Friedman, J. *FlowCytometryTools*. (Zenodo, 2015). doi:10.5281/ZENODO.596118
