# Supplementary material for: Improving prime editing with an endogenous small RNA-binding protein
Source: Nature. 2024 Apr 3;628(8008):639–47. doi: 10.1038/s41586-024-07259-6 (PMC11023932; doi:10.1038/s41586-024-07259-6)
Supplement: Supplementary file 2 — Reporting Summary [file 41586_2024_7259_MOESM2_ESM.pdf]

nature portfolio

Corresponding author(s): Britt Adamson

Last updated by author(s): Mar 21, 2024

Reporting Summary

Nature Portfolio wishes to improve the reproducibility of the work that we publish. This form provides structure for consistency and transparency in reporting. For further information on Nature Portfolio policies, see our Editorial Policies and the Editorial Policy Checklist.

Statistics

For all statistical analyses, confirm that the following items are present in the figure legend, table legend, main text, or Methods section.

n/a	Confirmed	
☐	☒	The exact sample size (*n*) for each experimental group/condition, given as a discrete number and unit of measurement
☐	☒	A statement on whether measurements were taken from distinct samples or whether the same sample was measured repeatedly
☐	☒	The statistical test(s) used AND whether they are one- or two-sided<br>*Only common tests should be described solely by name; describe more complex techniques in the Methods section.*
☐	☒	A description of all covariates tested
☐	☒	A description of any assumptions or corrections, such as tests of normality and adjustment for multiple comparisons
☐	☒	A full description of the statistical parameters including central tendency (e.g. means) or other basic estimates (e.g. regression coefficient) AND variation (e.g. standard deviation) or associated estimates of uncertainty (e.g. confidence intervals)
☐	☒	For null hypothesis testing, the test statistic (e.g. *F*, *t*, *r*) with confidence intervals, effect sizes, degrees of freedom and *P* value noted<br>*Give P values as exact values whenever suitable.*
☒	☐	For Bayesian analysis, information on the choice of priors and Markov chain Monte Carlo settings
☒	☐	For hierarchical and complex designs, identification of the appropriate level for tests and full reporting of outcomes
☐	☒	Estimates of effect sizes (e.g. Cohen's *d*, Pearson's *r*), indicating how they were calculated

Our web collection on statistics for biologists contains articles on many of the points above.

Software and code

Policy information about availability of computer code

Data collection	*Provide a description of all commercial, open source and custom code used to collect the data in this study, specifying the version used OR state that no software was used.*
Data analysis	Flow cytometry data were analyzed and FACS were performed with BD FACSDiva (8.0.1), Attune Cytometric Software (5.2.0), FlowCytometryTools (0.5.1, https://github.com/eyurtsev/FlowCytometryTools).<br><br>Genome-scale screen analyses used custom Python scripts (https://github.com/mhorlbeck/ScreenProcessing) with Python (2.7.18), CRISPhieRmix (0.1.0, https://github.com/timydaley/CRISPhieRmix), R (4.2.2), and ggplot2 (3.4.1).<br><br>Genome editing experiments used: HTSEQ (Princeton University High Throughput Sequencing Database, https://htseq.princeton.edu/), Cutadapt (4.1), and CRISPResso2 (2.2.11, https://github.com/pinellolab/CRISPResso2).<br><br>small RNA-seq analysis used: HTSEQ, a Snakemake (7.32.4) workflow, R (4.3.2), Cutadapt (4.1), Bowtie2 (2.5.0), a custom Python (3.11) script, Rsamtools (2.16.0), plyranges (1.20.0), DESeq2 (1.38.3), the apeglm algorithm (1.22.1), and ggplot2 (3.4.4) on data organized using the readr (2.1.4), dplyr (1.1.3), tidyr (1.3.0), and stringr (1.5.0) packages.<br><br>RNA-seq analysis used: HTSEQ, a Snakemake (7.32.3) workflow, R (4.3.1), STAR (2.7), DESeq2 (1.38), ashr package (2.2_54), R (4.3.1) with packages ggplot2 (3.4.3) and ggpubr (0.6.0).<br><br>Code for small RNA-seq analysis is available at https://zenodo.org/records/10553303 or https://github.com/Princeton-LSI-ResearchComputing/PE-small-RNA-seq-analysis. Code for RNA-seq analysis is available at https://zenodo.org/records/10553340 or https://

github.com/Princeton-LSI-ResearchComputing/PE-mRNA-seq-diffexp.

For manuscripts utilizing custom algorithms or software that are central to the research but not yet described in published literature, software must be made available to editors and reviewers. We strongly encourage code deposition in a community repository (e.g. GitHub). See the Nature Portfolio guidelines for submitting code & software for further information.

Data

Policy information about availability of data

All manuscripts must include a data availability statement. This statement should provide the following information, where applicable:

- Accession codes, unique identifiers, or web links for publicly available datasets
- A description of any restrictions on data availability
- For clinical datasets or third party data, please ensure that the statement adheres to our policy

GRCh38.p13 (GCA_000001405.28, PRJNA31257) from Ensembl release 107 used for small RNA-seq analysis is available at http://ftp.ensembl.org/pub/release-107/fasta/homo_sapiens/dna/Homo_sapiens.GRCh38.dna.primary_assembly.fa.gz.

GENCODE gene annotation release 43 used for small RNA-seq analysis is available at https://ftp.ebi.ac.uk/pub/databases/gencode/Gencode_human/release_43/gencode.v43.primary_assembly.annotation.gff3.gz.

GRCh38.p13 (GCA_000001405.28, PRJNA31257) from Ensembl release 100 used for RNA-seq is available at https://ftp.ensembl.org/pub/release-100/fasta/homo_sapiens/dna/Homo_sapiens.GRCh38.dna.primary_assembly.fa.gz.

High-throughput sequencing data of primary human T cell experiments have been deposited at GEO (GSE255003) and the NCBI Sequence Read Archive database under accession PRJNA1073019.

High-throughput sequencing data of primary human HSPC experiments have been deposited at the NCBI Sequence Read Archive database under accession PRJNA1071146.

All other high-throughput sequencing data have been deposited at GEO (GSE253424) and the NCBI Sequence Read Archive database under accession PRJNA1065772.

Research involving human participants, their data, or biological material

Policy information about studies with human participants or human data. See also policy information about sex, gender (identity/presentation), and sexual orientation and race, ethnicity and racism.

Reporting on sex and gender	*Use the terms sex (biological attribute) and gender (shaped by social and cultural circumstances) carefully in order to avoid confusing both terms. Indicate if findings apply to only one sex or gender; describe whether sex and gender were considered in study design; whether sex and/or gender was determined based on self-reporting or assigned and methods used. Provide in the source data disaggregated sex and gender data, where this information has been collected, and if consent has been obtained for sharing of individual-level data; provide overall numbers in this Reporting Summary. Please state if this information has not been collected. Report sex- and gender-based analyses where performed, justify reasons for lack of sex- and gender-based analysis.*
Reporting on race, ethnicity, or other socially relevant groupings	For T cells, Leukopaks were purchased independently of race, ethnicity, or other socially relevant groupings. For HSPCs, mobilized peripheral blood was obtained without information on race, ethnicity, or other socially relevant groupings.
Population characteristics	T cells were sourced from Leukopaks which were bought from Stemcell. The donors were chosen without regard to sex, gender, ethnicity or race. For HSPCs, see above.
Recruitment	Stemcell Technology recruits donors for Leukopak manufacturing. T cells were sourced from these Leukopaks using Stemcell Tecnologies magnetic isolation kits. De-identified CD34+ hematopoietic stem and progenitor cells (HSPCs) obtained following informed consent including for genomic testing, sharing and publication of de-identified data.
Ethics oversight	For T cell experiments, cells were obtained from StemCell (Stemcell Technologies, 200-0092) with approved Stemcell IRB. Use of deidentified cells is considered exempt human subjects research and is approved by UCSF IRB.<br><br>The CD34+ HSPCs used in this study were de-identified and research use consent had been previously obtained. Since the de-identified human specimens were not collected specifically for this study and our study team could not access any subject identifiers linked to the specimens or data, the Boston Children's Hospital (BCH) IRB has determined this is not considered human subjects research.

Note that full information on the approval of the study protocol must also be provided in the manuscript.

Field-specific reporting

Please select the one below that is the best fit for your research. If you are not sure, read the appropriate sections before making your selection.

☒ Life sciences ☐ Behavioural & social sciences ☐ Ecological, evolutionary & environmental sciences

Life sciences study design

All studies must disclose on these points even when the disclosure is negative.

Sample size	Sample sizes typical of genome-scale screens (PMID: 25307932) and genome editing experiments (PMID: 31634902) reported in the literature were used throughout. These sample sizes were sufficient to ensure data reproducibility while also maintaining experimental practicality.
Data exclusions	No data were excluded. (N/A)
Replication	CRISPRi screens were performed in independent biological duplicate. Small RNA sequencing and RNA sequencing experiments were performed in three or more independent biological replicates. Genome editing experiments were also performed in three or more independent biological replicates (except where indicated). Only experiments related to Extended data Fig. 1d, e, and f were performed in one replicate and cell line characterizations in Fig. 2a and Extended data Fig. 3d, e, g were performed in one replicate/on one defined cell line. All attempts at replication were successful.
Randomization	Experiments were designed to provide quantitative results with minimal subjective bias. Randomization was therefore not used. (N/A)
Blinding	Experiments were designed to provide quantitative results with minimal subjective bias. Blinding was therefore not used. (N/A)

Reporting for specific materials, systems and methods

We require information from authors about some types of materials, experimental systems and methods used in many studies. Here, indicate whether each material, system or method listed is relevant to your study. If you are not sure if a list item applies to your research, read the appropriate section before selecting a response.

Materials & experimental systems

n/a	Involved in the study	
☐	☒	Antibodies
☐	☒	Eukaryotic cell lines
☒	☐	Palaeontology and archaeology
☒	☐	Animals and other organisms
☒	☐	Clinical data
☒	☐	Dual use research of concern
☒	☐	Plants

Methods

n/a	Involved in the study	
☒	☐	ChIP-seq
☐	☒	Flow cytometry
☒	☐	MRI-based neuroimaging

Antibodies

Antibodies used	Anti-SSB antibody [mAbcam75927] (ab75927)<br>Recombinant Anti-GAPDH antibody [EPR16891] - Loading Control (ab181602)<br>Guide-it Cas9 Polyclonal Antibody (Takara 632607)<br>Anti-IgG Sheep Antibody (HRP (Horseradish Peroxidase)) (VWR 95017-332)<br>Anti-IgG Donkey Polyclonal Antibody (HRP (Horseradish Peroxidase)) (VWR 95017-556)
Validation	https://www.abcam.com/products/primary-antibodies/ssb-antibody-mabcam75927-ab75927.pdf<br>https://www.abcam.com/products/primary-antibodies/gapdh-antibody-epr16891-loading-control-ab181602.pdf<br>https://www.takarabio.com/documents/Certificate%20of%20Analysis/632607/632607-122314.pdf

Eukaryotic cell lines

Policy information about cell lines and Sex and Gender in Research

Cell line source(s)	Lenti-X 293T was purchased from Takara (632180). K562 (CCL-243), HeLa (CCL-2) and U2OS (HTB-96) were purchased from ATCC. K562 CRISPRi cell line was a gift from Jonathan Weissman.<br><br>Primary human T cells were isolated from human peripheral blood Leukopaks enriched for PBMCs sourced from STEMCELL Technologies (catalog # 200-0092) using the EasySep Human T cell isolation kit (STEMCELL Technologies 100-0695).<br><br>Cryopreserved human CD34+ HSPCs from mobilized peripheral blood of deidentified healthy donors were obtained from the Fred Hutchinson Cancer Research Center (Seattle, Washington), supported by Fred Hutch Cooperative Center of Excellence in Hematology (U54 DK106829).
Authentication	Lenti-X 293T (Takara 632180), K562 (CCL-243), HeLa (CCL-2), U2OS (HTB-96) and K562 CRISPRi cell line were authenticated by ATCC using STR analysis.

Mycoplasma contamination	All cell lines tested negative for mycoplasma.
Commonly misidentified lines (See ICLAC register)	No commonly misidentified cell lines were used in the study.

Flow Cytometry

Plots

Confirm that:

☒ The axis labels state the marker and fluorochrome used (e.g. CD4-FITC).

☒ The axis scales are clearly visible. Include numbers along axes only for bottom left plot of group (a 'group' is an analysis of identical markers).

☐ All plots are contour plots with outliers or pseudocolor plots.

☒ A numerical value for number of cells or percentage (with statistics) is provided.

Methodology

Sample preparation	For quantification of fluorescent protein expression or cell counting, suspension culture of K562 (ATCC CCL-243), K562 CRISPRi, and their derived cell lines were directly analyzed by flow cytometry.<br><br>For cell sorting, cells were counted by Countess or Attune NxT Flow Cytometer, pelleted by centrifugation at 200 g for 5 minutes, resuspended in DPBS supplemented with 10% FBS at 1-1.5E7 total cell/mL, and filtered by FACS tubes with cell strainer caps (Falcon 352235).
Instrument	Attune NxT Flow Cytometer or BD FACSAria Fusion Flow Cytometer was used for analysis of fluorescent protein expression and/or cell counting. BD FACSAria Fusion Flow Cytometer was used for cell sorting and sorting-related analysis of fluorescent protein expression.
Software	BD FACSDiva 8.0.1 Software was used to control BD FACSAria Fusion Flow Cytometer.<br>Attune Cytometric Software 5.2.0 was used to control Attune NxT Flow Cytometer.<br>BD FACSDiva 8.0.1, Attune Cytometric Software 5.2.0, and FlowCytometryTools 0.5.1 (eyurtsev.github.io/FlowCytometryTools/) were used to analyze flow cytometry data.
Cell population abundance	Cell numbers and/or relevant population abundances are reported in associated figure or Supplementary Table 7.
Gating strategy	For flow cytometry analyses and FACS, cells were first gated on FSC-A/SSC-A to exclude debris and then gated on FSC-A/FSC-H to select single cells. Cells were then gated on one or more transgene markers (mCherry, BFP, and/or GFP). For cell viability analysis or cell counting, only the FSC-A/FSC-H gate was used. For more detailed information and example gating strategies refer to Supplementary Information.

☒ Tick this box to confirm that a figure exemplifying the gating strategy is provided in the Supplementary Information.
